# Behavioral manifestations and underlying mechanisms of amphetamine in constructing animal models of mania: a comprehensive review

**DOI:** 10.3389/fnins.2025.1544311

**Published:** 2025-05-09

**Authors:** Zi-Qi Deng, Xiao-Chen Si, Jia-Bin Song, Jin-Yao Li, Lu Sun, Xue Dang, Min Zhao, Yan-Chen Feng, Fei-Xiang Liu

**Affiliations:** ^1^Department of Neuropsychiatric Psychology, Hospital of Encephalopathy, The First Affiliated Hospital of Traditional Henan University of Chinese Medicine, Zhengzhou, China; ^2^Institute of Management and Science University, Henan University of Chinese Medicine, Zhengzhou, China; ^3^Traditional Chinese Medicine (Zhong Jing) School, Henan University of Chinese Medicine, Zhengzhou, China; ^4^College of Acupuncture, Moxibustion and Tuina, Henan University of Chinese Medicine, Zhengzhou, China; ^5^The First Clinical Medical School, Henan university of Chinese Medicine, Zhengzhou, China

**Keywords:** mania, amphetamine, neurotransmitter, signal pathway, animal mode

## Abstract

Mania is a mind disorder with heightened emotions, etc. Amphetamine (AMPH), a drug with central nervous system excitatory effects, can disrupt neurotransmitter release and metabolism, causing mania. AMPH-induced animal models of mania show increased risk and reward-seeking behaviors and excessive locomotion like mania patients, verifiable by tests like Elevated Plus Maze (EPM). It also impacts neurotransmitter balance in different brain regions, aligning with the imbalance in mania patients. Multiple signaling pathways including extracellular regulated protein kinases and others are involved, and their altered activities link to mania symptoms. In the AMPH-induced mania model, regions like the frontal cortex have increased oxidative stress and inflammatory response. Moreover, AMPH changes neurotrophin levels, potentially causing neuronal damage and cognitive impairment. In summary, the AMPH-induced mania animal model is crucial for studying mania’s pathogenesis. However, further in-depth studies on neurotransmitter regulation, signaling pathway intervention, and neurotrophic factors are needed to develop more effective and personalized treatment plans.

## 1 Introduction

Mania, a psychological disorder condition predominantly often seen during the pre-phase of bipolar spectrum disorders (Daniels and Boison, 2023), presents with a distinct constellation of clinical features. These include elevated mood, psychomotor agitation, inflated self-esteem, pressured speech, cognitive disorganization, and high-risk behavioral patterns, frequently accompanied by reduced sleep requirements and emotional lability (Keramatian et al., 2022). Epidemiological surveillance data reveal a 2.9% prevalence rate among adolescents, demonstrating significant gender disparity with female predominance (3.3 vs. 2.6% in males) (Clemente et al., 2015). Contemporary etiological models conceptualize mania as a multifactorial convergence disorder arising from dynamic interactions between genetic predisposition, psychosocial stressors, and environmental determinants (Tondo et al., 2017). Notably, circadian rhythm dysregulation (particularly chronic sleep deprivation), acute psychosocial stressors, and substance use disorders (including nicotine dependence) have been identified as critical precipitating factors modulating disease trajectory (Dubovsky, 2015). Genomic investigations have unveiled substantial polygenic overlap with schizophrenia and major depressive disorder, sharing multiple risk-conferring single nucleotide polymorphisms (SNPs) across these diagnostic entities (Mullins et al., 2021). This complex pathophysiology leads to multiple health threats for people with mania, with suicide risk being particularly prominent. Cross-national cohort studies document annualized suicide mortality rates of 200-400 per 100,000 person-years—exceeding general population rates by over 30-fold. Clinically, 4-19% of affected individuals succumb to completed suicide, while 20-60% exhibit at least one documented suicide attempt during disease progression (Maniglio, 2013), underscoring the imperative for early detection and preventive intervention. Recent advances in neurobiological research have elucidated multi-level mechanistic disturbances underlying manic pathophysiology. These include: Monoaminergic dysregulation (particularly dopaminergic and serotonergic systems) at the molecular level. Intracellular signaling anomalies involving secondary messenger cascades (cAMP, PI3K/Akt/mTOR pathways) impacting neuroplasticity; Systemic alterations encompassing oxidative stress imbalance and aberrant neurotrophic factor expression (e.g., BDNF) (Ferrari et al., 2011; Yang et al., 2023). These multilayered discoveries not only refine our nosological understanding of bipolar spectrum disorders but also provide a mechanistic framework for developing targeted therapeutic modalities.

The development of reliable animal behavioral models remains a persistent challenge in mania research, significantly impeding investigations into its neurobiological underpinnings and the discovery of novel therapeutic agents. Contemporary modeling paradigms principally comprise three methodological approaches: pharmacological induction, genetic modification, and physical manipulation (Lopachev et al., 2019). Pharmacologically induced models have gained prominence in both mechanistic studies and pharmacological screening due to their operational feasibility, cost-effectiveness, and protocol standardization advantages (Steckert et al., 2013). Compared to genetic models, pharmacologically induced paradigms demonstrate superior capacity for recapitulating the clinical phenomenology of mania, while offering enhanced mechanistic specificity and experimental reproducibility relative to physical induction methods—characteristics that render them particularly suitable for interrogating drug mechanisms and intracellular signaling cascades (Verharen et al., 2023; Li et al., 2023). Nevertheless, these models exhibit notable limitations including inter-individual variability in behavioral responses and inconsistent phenotypic stability across experimental iterations.

Amphetamine (AMPH), a prototypical central nervous system stimulant, exists as a racemic mixture comprising dextrorotatory (d-) and levorotatory (l-) enantiomers. The d-isomer (d-AMPH) elicits aberrant monoaminergic transmission through selective inhibition of dopamine transporter (DAT) function—a pathomechanism demonstrating remarkable congruence with postmortem findings of striatal DAT downregulation in manic patients, thereby providing robust empirical support for dopamine hyperactivity hypotheses in this disorder (van Enkhuizen et al., 2015b). Structural modification of AMPH via methyl substitution at the benzene ring’s 3-carbon position yields methamphetamine (m-AMPH), which exhibits enhanced lipophilicity and blood-brain barrier permeability. While m-AMPH effectively replicates oxidative stress biomarkers characteristic of manic neuropathology, its induction of mitochondrial dysfunction and neuroinflammatory cascades has been associated with elevated experimental mortality rates, substantially constraining its utility in longitudinal studies (Kanazawa et al., 2021). In contrast, the AMPH derivative 3,4-methylenedioxymethamphetamine (MDMA) primarily targets serotonin transporters (SERT), exhibiting greater pharmacological alignment with 5-hydroxytryptamine (5-HT)-related psychopathology and consequently reduced applicability in modeling manic states (Chaves et al., 2020). Current evidence positions AMPH-based models as the pharmacological gold standard for mania induction, owing to their superior fidelity in replicating core symptomatology and neurobiological signatures (Hsueh et al., 2021; Johnson et al., 2023). Alternative inducers such as ouabain demonstrate limited translational value due to inadequate simulation of neurotransmitter dynamics and region-specific neuropathological alterations (Vargas et al., 2006). Notably, AMPH models present methodological challenges including pharmacokinetic variability and dependence liability. These limitations may be mitigated through systematic implementation of dose-response curve optimization, refined administration protocols, and multimodal behavioral phenotyping incorporating open-field, EPM, and Forced Swim Test paradigms. Complementary integration of molecular neuroimaging techniques could further elucidate striatal-prefrontal circuit dysfunction, thereby enhancing the model’s mechanistic interpretability (Kanazawa et al., 2021).

The development of medical translational value animal models of mania necessitates rigorous evaluation across three core validation domains: Face Validity (operationalized through behavioral paradigms including open-field testing and stereotypy quantification), Construct Validity (anchored in pathophysiological biomarkers such as striatal dopamine transporter density measurements and cAMP pathway profiling), and Predictive Validity (substantiated by therapeutic responses to established mood stabilizers including lithium and valproate) (Brandon et al., 2003; Chaves-Filho et al., 2024). This comprehensive review synthesizes methodological advancements since 1970, with particular emphasis on behavioral phenotyping, neurotransmitter changement, and critical intracellular signaling pathway in AMPH animal models, aiming to establish a mechanistic framework for new therapeutic development (Guimarães et al., 2024).

## 2 Methods for AMPH-induced mania model

### 2.1 Acute vs. chronic dosing regimens

The critical operational parameters in AMPH-induced mania model encompass dosage regimen, administration frequency, and treatment duration (Table 1). Acute induction protocols predominantly employ single intraperitoneal (i.p.) injections of 2.5-4.0 mg/kg (in 0.9% saline vehicle), a dose range empirically established as inducing robust psychomotor hyperactivity and novelty-seeking behaviors while maintaining absence of significant toxicological manifestations (Dalby-Brown et al., 2013). Chronic modeling paradigms employ extended 10-day i.p. regimens (once-daily administration within a fixed temporal window of 09:00-11:00 h), with sustained exposure recapitulating key manic phenotypes including progressive escalation of stereotypy indices from day 7 post-initiation and concomitant circadian rhythm desynchronization (Phan et al., 2020).

**TABLE 1:** Summary of parameters of different modeling methods for the AMPH induction model.

Parameter	Acute modeling	Chronic modeling	IVSA paradigm
Dose range	2.5-4.0 mg/kg	2.5-3.5 mg/kg/day	0.05 mg/kg/infusion
Administration frequency	Single dose	q.d. × 10 days	Self-triggered (≤50 infusions/day)
Peak time	30 ± 5 min	28 ± 3 min	Immediate
Behavioral window	30-90 min post-administration	24 h post-last dose	Entire training period
Application	Pharmacodynamic screening	Disease progression simulation	Addiction liability assessment
References	Dalby-Brown et al., 2013	Phan et al., 2020	Hadamitzky et al., 2011

### 2.2 Route of administration specificity parameter

Administration route selection profoundly modulates the drug’s pharmacokinetic profile (Table 2). The classical intraperitoneal (i.p.) method employs AMPH solution delivered at 5 mL/kg, demonstrating peak plasma concentrations at 30 ± 5 min post-injection with pharmacodynamic effects persisting 4-6 h (Chaves-Filho et al., 2024). Comparatively, intravenous self-administration (IVSA) paradigms enable rodents to autonomously acquire 0.05 mg/kg/dose (0.1 mL/s infusion rate) through operant conditioning, exhibiting progressive escalation of daily intake from 0.8 ± 0.2 to 3.4 ± 0.5 mg/kg over 10 training days—a pattern effectively recapitulating substance use disorder progression (Hadamitzky et al., 2011).

**TABLE 2:** Summary of modeling parameters relevant to AMPH induction models.

Animal strain	Age/weight	Administration route	Dosage and regimen	References
C57BL/6 mice	10–12 weeks old, male	Intraperitoneal (i.p.)	2.5 mg/kg, 10 consecutive days	Li et al., 2023
C57BL/6 mice	8 weeks old, male, 24 ± 5 g	Intraperitoneal (i.p.)	2.5 mg/kg, 10 consecutive days	Phan et al., 2020
C57BL/6J mice	6–8 weeks old, male	Intraperitoneal (i.p.)	4 mg/kg, single injection	Dalby-Brown et al., 2013
Swiss mice	25–30 g, male	Intraperitoneal (i.p.)	0.1 mL/10 g body weight	Chaves-Filho et al., 2024
Adult albino swiss mice	30–45 g, male	Intraperitoneal (i.p.)	1.0, 2.0, or 3.0 mg/kg, undefined regimen	Vargas et al., 2006
Sprague dawley rats	350–450 g, adult male	Intravenous self-administration (IVSA)	0.05 mg/kg/infusion, gradual protocol extension (1 h/day → 3 h/day → 6 h/day), 6 days per phase, 2-day abstinence intervals	Hadamitzky et al., 2011

### 2.3 Dose differences induce different behavioral differences

Low-dose regimens (≤ 4 mg/kg) preferentially elicit core manic symptomatology including psychomotor hyperactivity, reduced sleep requirement, and enhanced novelty-seeking behavior, demonstrating optimal suitability for preliminary pharmacological screening studies (Table 2). In contrast, high-dose administration (5-8 mg/kg) provokes not only intensified stereotypies but also neurochemical perturbations characterized by cerebellar GABAergic dysregulation and prefrontal cortex NE/5-HT ratio imbalance—phenomena requiring verification through region-specific *in vivo* microdialysis methodologies (Yin et al., 2010).

### 2.4 Ethical stuff

All experimental procedures were conducted in compliance with AAALAC-accredited specific pathogen-free (SPF) facility standards. Animal housing maintained controlled environmental parameters: temperature 22 ± 1°C, relative humidity 60 ± 5%, 12-h light/dark cycle (lights on at 06:00 h), social housing density (≤ 5 subjects per cage), and weekly replacement of autoclaved aspen chip bedding. Ethical safeguards implemented included: confinement of experimental durations to ≤ 14-day cycles, incremental dosing protocols (≤ 0.5 mg/kg daily escalation), and continuous biometric surveillance (≤ 20% maximum allowable body weight reduction), with all operational protocols undergoing mandatory IACUC review and approval (Brandon et al., 2003).

## 3 Behavioral tests for locomotor activity

### 3.1 Open Field Test

The Open Field Test (OFT), a cornerstone behavioral paradigm for evaluating manic-like psychomotor hyperactivity and disinhibited exploration, operationalizes the clinical phenomenon of impaired risk assessment in mania through quantification of novel environment exploration patterns (Valvassori et al., 2022). Experimental implementation involves placing subjects in standardized acrylic box (50 × 50 × 50 cm with defined central zone occupying 33% surface area) for 5-min video-tracked sessions.

Core behavioral metrics focus on spatial exploration bias, with AMPH-induced mania model exhibiting significantly elevated central zone occupancy—a behavioral proxy validating face validity through replication of human manic risk-taking phenotypes (Ene et al., 2016; Souza et al., 2016). Lithium carbonate administration demonstrates predictive validity by normalizing hyperlocomotor activity (Ackermann et al., 2010; Han et al., 2018). Neurochemically, this behavioral dysregulation correlates with striatal dopamine transporter (DAT) downregulation-induced extracellular DA accumulation and concomitant prefrontal choline acetyltransferase (ChAT) suppression, mechanistically substantiating the catecholaminergic-cholinergic imbalance hypothesis of bipolar disorder while establishing construct validity (van Enkhuizen et al., 2015a).

While OFT remains the methodological gold standard due to its semi-automated quantification advantages, interpretational limitations persist regarding environmental confounders (Çiçekli et al., 2022; Steckert et al., 2013). Current best practices recommend multimodal behavioral profiling incorporating complementary paradigms: EPM for risk-preference stratification, bite force assays (BFT) for aggression quantification, and resident-intruder tests for impulse control evaluation, collectively enhancing translational relevance through multidimensional behavioral characterization (Table 3).

**TABLE 3:** Behavioral testing of animal models of mania introduction to organizing.

Behavioral test	Evaluation metrics	Results	Advantages	Limitations	Related neural mechanisms
OFT (Open Field Test)	Central zone exploration time, locomotor trajectory	AMPH group shows increased central exploration, reversible by lithium	Semi-automated analysis, gold-standard validation	Susceptible to environmental confounders	Striatal DAT downregulation, prefrontal ChAT suppression
FST (Forced Swim Test)	Immobility duration	AMPH group exhibits reduced immobility, reversible by valproate	Operational simplicity, high-throughput potential	Immobility reduction may reflect environmental adaptation	Prefrontal 5-HT efflux elevation, 5-HT1A receptor activation
TST (Tail Suspension Test)	Struggle duration	AMPH group demonstrates prolonged late-phase struggle, reversible by lithium/valproate	Rapid procedure, high-throughput capacity	Forelimb strength variability confounds results, insensitive to anxiety phenotypes	VTA DA neuron firing potentiation, PVN CRH mRNA upregulation
EPM (Elevated Plus Maze)	Open-arm entries, open-arm time ratio	AMPH group displays increased open-arm exploration, reversible by diazepam	High efficiency	Apparatus size variability, locomotor interference	Prefrontal 5-HT imbalance, GABAA receptor phosphorylation anomalies
SPT (sucrose preference test)	Sucrose preference ratio (sucrose intake/total intake × 100%)	AMPH group shows elevated preference, reversible by lithium	Most convenient hedonic assessment	Hyperactivity-induced “pseudo-anhedonia” artifacts	Midbrain limbic DA circuit hyperactivation
TCST (Three-Chamber Social Test)	Stranger zone duration, trajectory entropy	AMPH group exhibits social hyperactivation, reversible by lithium/valproate	Visualizes social motivation	“Social hyperactivation” may reflect exploration bias	DA D2 receptor signaling enhancement, prefrontal-amygdala dysconnectivity
BFT (Bite Force Test)	Crushed substrate weight	AMPH group demonstrates enhanced bite force, reversible by valproate	Simple operation, high reproducibility	Biting may reflect foraging instincts	Striatal DA elevation, muscle fiber ATP synthesis acceleration
PIST (Pentobarbital Induced Sleep Test)	Sleep latency, Sleep duration	AMPH group displays reduced deep sleep, reversible by valproate	Direct sleep state measurement	Pharmacologically induced vs. natural sleep differences	Prefrontal ATP turnover acceleration, GABAergic system alterations

### 3.2 Forced Swim Test

The Forced Swim Test (FST) serves as a critical behavioral assay for evaluating despair-like behavioral dysregulation in mania models, with its theoretical framework rooted in the prefrontal-limbic circuitry dysregulation hypothesis underlying manic hyperactivity (Lee et al., 2017). Experimental implementation utilizes transparent polycarbonate cylinders (10 cm diameter × 25 cm height) containing 8 cm depth of temperature-controlled water (24°C ± 1°C). Behavioral assessment is based on the temporal transition from escape-oriented struggling during the first 2-min to behavioral quiescence in the subsequent 4-min period, with immobility duration serving as the key parameter for quantifying behavioral inhibition in constrained environments (Bogdanova et al., 2013).

AMPH-induced mania model demonstrate significantly attenuated immobility durations versus controls, while valproate sodium intervention restores immobility parameters to normative ranges without compromising locomotor capacity—findings that collectively validate the paradigm’s predictive validity for therapeutic screening (Flaisher-Grinberg and Einat, 2009). Construct validity is further substantiated through neurochemical parallels: AMPH-treated subjects exhibit elevated extracellular 5-HT levels, mirroring postmortem findings of reduced dorsal raphe nucleus 5-HT neuronal density in manic patients, coupled with prefrontal 5-HT1A receptor upregulation (van Enkhuizen et al., 2015b).

Mechanistically, AMPH-induced prefrontal serotonergic efflux activates 5-HT1A autoreceptor-mediated negative feedback modulation, thereby suppressing despair-related behavioral manifestations through cortico-limbic pathway regulation (Trunnell and Carvalho, 2021).

### 3.3 Tail Suspension Test

The Tail Suspension Test (TST) constitutes a validated behavioral paradigm for assessing disinhibited motivational states in manic models, with neurobiological rationale rooted in prefrontal-amygdala circuit dysfunction underlying impulse dysregulation in bipolar mania. Experimental implementation involves securing rodents in dedicated tail suspension apparatus (15 cm ground clearance) for 6-min sessions with continuous quantification of struggle intensity and immobility duration. Unlike water-based FST paradigms, TST induces behavioral despair through mechanical stress induction, with active struggle duration serving as a key metric of stress coping capacity.

AMPH-induced mania model exhibited prolonged struggle persistence during late-phase testing (4-6 min), demonstrating behavioral homology to human manic impulsivity in clinical populations (Cryan et al., 2005). Mood stabilizers (lithium carbonate/sodium valproate) dose-dependently normalized immobility parameters, confirming pharmacological predictive validity (Yan et al., 2010). Neurochemical validation revealed dopaminergic hyperactivity in model animals, evidenced by elevated CSF homovanillic acid (HVA) levels and circadian cortisol rhythm disruption—biochemical signatures mirroring clinical manic states (van Enkhuizen et al., 2015a). Mechanistically, acute AMPH exposure potentiates ventral tegmental area (VTA) dopaminergic firing via D1 receptor-mediated motor drive enhancement, while chronic administration induces hypothalamic paraventricular nucleus (PVN) CRH mRNA upregulation, indicating hypothalamic-pituitary-adrenal (HPA) axis feedback dysregulation (Siefried et al., 2020).

Despite operational advantages (10-min protocol; 6-animal parallel testing capacity), TST interpretation requires caution due to confounders including forelimb strength variability and insensitivity to anxiety/cognitive domains. Emerging protocols recommend synergistic use with EPM to dissect anxiety-struggle time interactions, potentially enhancing specificity for manic motivational circuitry assessment.

### 3.4 Elevated Plus Maze Test

The Elevated Plus Maze (EPM) serves as a critical behavioral paradigm for evaluating anxiety-impulsivity interactions in mania models, grounded in the neurobiological premise of prefrontal-amygdala neural circuitry dysfunction underlying risk assessment abnormalities in bipolar disorder (Pentkowski et al., 2021). The standardized apparatus comprises two open arms (25 × 5 cm) and two enclosed arms (identical dimensions with 15 cm high walls) intersecting at a neutral central platform (5 × 5 cm). Experimental protocol initiates with subject placement on the central zone facing enclosed arms, followed by 5-min video tracking of exploratory patterns. Risk-preference quantification employs open-arm time ratio versus enclosed-arm dwell time as primary behavioral indices (Cho et al., 2022; Heinz et al., 2021).

AMPH-induced mania model demonstrated pathological behavioral signatures: increased open-arm entries and elevated open-arm time percentage, with this disinhibition profile exhibiting synergistic correlation with OFT central zone preference—collectively establishing face validity (Leem et al., 2023). Pharmacological validation via diazepam-induced normalization of open-arm exploration parameters confirms the paradigm’s predictive validity (Komada et al., 2008). Molecular construct validity emerges through serotonergic dysregulation mirroring clinical pathology: reduced nucleus accumbens 5-HT neuronal density and prefrontal mitochondrial complex inhibition in model animals recapitulate postmortem manic patient findings (Hao et al., 2019). Mechanistically, these behavioral aberrations correlate with impaired GABAergic neurotransmission via altered GABA_*A*_ receptor phosphorylation states (Yoo et al., 2017).

Despite the test methodological efficiency (< 8-min/animal testing duration), critical limitations require consideration: confounding factors including interspecies apparatus scaling requirements and locomotor capacity interference necessitate rigorous standardization. Current optimization strategies propose multivariate behavioral phenotyping strategy integrating EPM with OFT parameters, enabling computational discrimination of manic-specific disinhibition from non-specific hyperactivity through discriminant function analysis.

### 3.5 Sucrose Preference Test

The Sucrose Preference Test (SPT) serves as a critical behavioral metric for evaluating reward processing dysregulation in manic models, anchored in the core pathophysiology of midbrain limbic dopaminergic circuit hyperactivation driving aberrant reward anticipation in bipolar mania (Primo et al., 2023). Experimental implementation employs a two-bottle free-choice paradigm: following 24-h habituation, subjects receive *ad libitum* access to 2.5% sucrose solution versus purified water (bottle weights recorded to ± 0.1 g precision). Reward-seeking motivation is quantified via dark cycle consumption-derived preference ratio [(sucrose intake/total fluid intake) × 100%] during 12-h testing windows (Isozaki et al., 2016).

AMPH-induced mania model exhibited pathological hyperhedonia, demonstrating significantly elevated sucrose preference ratios that neurobiologically correlate with nucleus accumbens hyperactivity patterns observed in manic patients during reward tasks—establishing robust face validity. Lithium carbonate administration selectively normalized preference indices without affecting basal consumption, confirming pharmacological predictive validity (Liu et al., 2022). Mechanistically, dopaminergic dysregulation induces ventral striatal reward threshold downregulation, driving maladaptive reward-seeking behaviors through mesolimbic pathway sensitization (Verharen et al., 2023).

While SPT remains the most operationally efficient paradigm for assessing reward processing, methodological caveats necessitate cautious interpretation. AMPH-induced locomotor hyperactivity may artifactually fragment feeding patterns, potentially generating confounding “False anhedonia” phenotypes. Current optimization protocols recommend concurrent pentobarbital sleep modulation to dissociate motivation-driven sucrose preference from non-specific consummatory behaviors through controlled feeding window analysis (Chestnykh et al., 2023).

### 3.6 Three-Chambered Sociability Test

The Three-Chambered Sociability Test (TCST) offers a visual assessment platform for evaluating aberrant social motivational patterns and cognitive deficits in manic models (Arakawa, 2023). This methodology is theoretically grounded in the impaired social distance perception resulting from disrupted prefrontal-amygdala functional connectivity during manic episodes (Hellvin et al., 2013). The standardized apparatus consists of three interconnected compartments (5 × 5 cm central zone and two 15 × 15 cm lateral zones), with a non-contact conspecific stimulus placed in the novel side compartment during testing. A video-tracking system records exploratory behavior parameters over a 10-min period, including time spent in the novel zone, contact frequency, and movement trajectory entropy, to quantify rodent social stimulus preference.

AMPH-induced mania model demonstrated paradoxical social hyperactivity characterized by prolonged novel zone residence and increased trajectory entropy, which contrasts with the social withdrawal typically observed in mania patients. While lithium and valproic acid interventions normalized these metrics—validating the test’s predictive validity—and *in vivo* dopamine D2 receptor signaling augmentation in AMPH models supported structural validity (Li et al., 2023), critical analysis of face validity is required. The observed “social hyperactivity” likely reflects non-specific exploratory behavior or drug-induced euphoria rather than genuine social competence.

Although face validity remains to be fully established, the demonstrated correlation between these behaviors and brain 5-HT levels suggests this test could advance serotonergic research in mania (Ahern et al., 2009). Integration with the EPM is recommended to concurrently validate face validity and investigate 5-HT’s mechanistic role in manic symptomatology.

### 3.7 Bite Force Test

The Bite Force Test (BFT) serves as a validated behavioral paradigm for quantifying mania-related aggression phenotypes, based on the clinical observation of heightened verbal/physical aggression in manic patients due to behavioral disorganization (Beentjes et al., 2012). This assay employs standardized apple twigs (uniform shape/size/weight) to quantify aggression via crumb weight measurement, operationalizing manic irritability in rodent models (Mitchell et al., 2024).

AMPH-induced mania model exhibited significant bite force augmentation—representing hyperaggressive behavior—that is reversed by valproate, demonstrating both face and predictive validity (Bartholomew, 1994). Mechanistically, AMPH exposure induces striatal dopamine (DA) hyperactivity concurrent with enhanced myofiber ATP synthesis, establishing neurochemical-metabolic coupling alterations consistent with postmortem findings of basal ganglia DA hypersensitivity (D2 receptor mRNA upregulation) in manic patients (Mannangatti et al., 2018). This dopaminergic hyperactivity directly mediates the observed phenotype through AMPH’s agonistic effects on DAergic signaling.

While the BFT offers simplicity and reproducibility through standardized twig provision and crumb weighing, interpretation requires caution. Elevated bite force may reflect non-specific behaviors (exploration, foraging, anxiety) rather than direct irritability. Multidimensional validation integrating anxiety assessment via the EPM is recommended to disambiguate motivational components.

### 3.8 Pentobarbital Sleep Experiment Test

The Pentobarbital Induced Sleep Test (PIST) represents a validated behavioral pharmacological paradigm for evaluating mania-related sleep dysregulation, capitalizing on the cardinal clinical feature of reduced sleep need/maintenance in manic patients (Beentjes et al., 2012). This assay involves intraperitoneal administration of pentobarbital diluted in 0.9% saline, with sleep latency and duration measured to characterize sleep architecture in rodent models (Egashira et al., 2021; Morton et al., 2021).

AMPH-induced mania model exhibited a phenotype of reduced non-rapid eye movement (NREM) sleep and augmented rapid eye movement (REM) sleep—mirroring clinical manic sleep profiles—that validates face validity. Valproate-mediated normalization of sleep duration further supports predictive validity (Cotovio and Oliveira-Maia, 2022). Mechanistically, AMPH exposure induces mitochondrial hyperactivity in the prefrontal cortex, manifesting as elevated ATP turnover and complex I-IV activity. This metabolic hyperactivation corresponds to postmortem findings of increased temporal lobe glucose metabolism in manic patients, establishing metabolic-level structural validity. Pathophysiological changes arise from AMPH-induced GABAergic dysfunction, specifically reductions in prefrontal GABA content (Yoo et al., 2017).

Notwithstanding its utility, PIST has caveats: pentobarbital induces pharmacological sleep via CNS depression, differing from endogenous sleep regulation. Thus, while directly quantifying sleep parameters, interpretations should consider potential dissociation from natural sleep mechanisms. Nevertheless, this assay provides novel insights into GABAergic involvement in mania and serves as a platform for translational sleep research (Table 3).

## 4 Neurotransmitter alterations

### 4.1 Dopamine

The neurobiological underpinnings of mania are inextricably linked to dopaminergic system dysregulation. Anatomically, dopaminergic projections originating in the ventral tegmental area innervate critical limbic and cortical regions including the hippocampus, striatum, nucleus accumbens, and prefrontal cortex, modulating motor control, emotional processing, and reward circuitry via interactions with G-protein coupled receptors (Zald, 2023; Miyake et al., 2011). Clinical pharmacology provides bidirectional evidence supporting this relationship: dopamine agonists like AMPH precipitate symptom exacerbation across the mania spectrum from euphoria and hypomania to psychotic mania by enhancing synaptic dopamine (DA) availability (Bari and Robbins, 2013), while D2 receptor antagonists—hallmark of second-generation antipsychotics—effectively attenuate acute manic symptoms, validating the therapeutic relevance of dopaminergic hyperactivity (Demontis et al., 2017). This dopaminergic hypothesis is further supported by neuroimaging studies demonstrating increased DA synthesis capacity and receptor supersensitivity in key brain regions of manic patients (Volkow et al., 2019).

Neurobiochemical investigations demonstrate characteristic elevation of central DA concentrations during manic episodes (Young et al., 2011). Rodent model studies reveal multi-dimensional regulatory mechanisms underlying AMPH-induced DA elevation in key brain regions including the striatum, prefrontal cortex, and hippocampus (Engelhardt et al., 2018; Støier et al., 2023). At the presynaptic level, AMPH disrupts mitochondrial energy metabolism by inhibiting tricarboxylic acid cycle enzymes (e.g., citrate synthase, malate dehydrogenase), reducing ATP synthesis and promoting vesicular DA release (Valvassori et al., 2013). Concurrently, endogenous cardiac steroids inhibit Na^+^/K^+^-ATPase, inducing intracellular Ca^2+^ overload that activates ERK signaling and mitochondrial KATP channel opening. This leads to reactive oxygen species (ROS)-mediated DA release through a redox-sensitive mechanism (Frey et al., 2006b; Hodes et al., 2018). Transporter regulation involves protein kinase C (PKC)-mediated phosphorylation of the DA transporter (DAT) N-terminus, causing conformational changes that abrogate reuptake function (Carvalho Pinheiro et al., 2012). Metabolic inhibition occurs when neurotransmitters like serotonin compete with monoamine oxidase (MAO) for binding sites, reducing DA conversion to DOPAC. This process is genetically modulated by MAO polymorphisms (Gubert et al., 2020; Rippberger et al., 2015; Shin et al., 2019; Sun et al., 2021). Pathophysiological consequences arise from synergistic D2 receptor hypersensitivity in the mesolimbic system (Varela et al., 2024) and D1 receptor dysfunction in the prefrontal cortex (Goloncser et al., 2024). Spatiotemporal differences in DA signaling may explain clinical heterogeneity: limbic DA hyperactivity correlates with euphoria, while prefrontal DA imbalance contributes to cognitive deficits. These integrated mechanisms provide a framework for understanding the neurochemical basis of manic symptomatology.

DA system dysregulation being established as a cardinal biological marker of mania, translating this finding into clinical practice remains challenging. Accumulating evidence indicates that monoamine concentration fluctuations occur not only in bipolar disorder but also in conditions like attention-deficit/hyperactivity disorder (Rihmer et al., 2017). This necessitates integrative diagnostic approaches incorporating neuroimaging signatures, receptor sensitivity profiling, and transcriptomic biomarkers. Future research should focus on elucidating the molecular determinants of DA signaling specificity, particularly the role of phosphorylation cascades such as DARPP-32 in mediating mood state switching (da-Rosa et al., 2012). Understanding how spatiotemporal variations in DAergic signaling contribute to symptom heterogeneity may lead to precision medicine strategies targeting discrete neurocircuitry abnormalities.

### 4.2 Glutamate

Dysfunction of the glutamatergic system is increasingly recognized as a critical pathological mechanism underlying manic episodes in bipolar disorder. Anatomically, glutamatergic projections densely innervate limbic structures including the hippocampus, striatum, and nucleus accumbens, where they regulate cognitive flexibility, emotional valence encoding, and synaptic plasticity through ionotropic (iGluR) and metabotropic (mGluR) receptor subtypes (Qiu et al., 2019).

Clinical neuroimaging studies reveal significantly increased glutamate (Glu) uptake in brain limbic structures of manic patients compared to healthy controls (Daniele et al., 2012), a phenomenon likely reflecting compensatory glial clearance of synaptic Glu overload. Longitudinal investigations demonstrate state-independent alterations in Glu transporter activity: individuals with bipolar disorder exhibit stable Glu uptake patterns regardless of clinical phase (Scotti-Muzzi et al., 2021), suggesting potential as an endophenotypic biomarker. Neurochemical analyses show elevated striatal and nucleus accumbens glutamine levels in mania, accompanied by augmented hippocampal astrocytic glutathione synthesis (Pereira et al., 2021; Shoblock et al., 2003). This indicates hyperactivation of the glial-neuronal glutamine cycle may provide excessive precursors for Glu synthesis. Current mechanistic validation in animal models remains limited, necessitating speculative frameworks for future research. Hyperactivation of α-amino-3-hydroxy-5-methyl-4-isoxazolepropionic acid receptors (AMPARs) and kainate receptors (KARs) drives neuronal hyperexcitability via BDNF-TrkB signaling, with dose-dependent correlations to manic agitation (Yankelevitch-Yahav et al., 2015). Additionally, regional cerebral vasoconstriction-induced hypoxia impairs Na^+^/K^+^-ATPase function by reducing ATP availability (Noda, 2016). This dual impairment disrupts Glu reuptake and reverses excitatory amino acid transporter (EAAT) directionality, leading to synaptic Glu accumulation.

Despite the positive correlation between Glu concentrations and manic symptom severity (Gubert et al., 2020), translating glutamatergic biomarkers into clinical practice faces dual challenges. Existing methodologies struggle to distinguish contributions from intracellular and extracellular Glu pools, while glutamatergic dysregulation overlaps significantly with anxiety disorders, schizophrenia, and other neuropsychiatric conditions (Shoblock et al., 2003). Future research should prioritize mechanistic dissection of regional Glu homeostasis using gene-edited animal models and development of subtype-specific interventions targeting excitatory amino acid transporters (EAATs) or metabotropic glutamate receptor (mGluR) allosteric modulators. Resolving spatiotemporal specificity of glutamatergic signaling is critical for differentiating bipolar mania from phenotypically similar disorders. Advancing this field requires integrating multi-omic datasets with circuit-level electrophysiology to map Glu dynamics in real-time.

### 4.3 γ-aminobutyric acid

Hypofunction of the γ-aminobutyric acid (GABA)ergic system represents a critical neurobiological substrate for manic episodes (Valvassori et al., 2008). Anatomically, GABAergic interneurons densely populate cortico-limbic circuits including the prefrontal cortex, hippocampus, and nucleus accumbens, where they maintain excitatory-inhibitory balance via GABA_*A*_ receptors (mediating fast synaptic inhibition) and GABA_*B*_ receptors (regulating slow synaptic transmission) (Petroff, 2002; Hsueh et al., 2021).

Clinical biomarker studies demonstrate reduced cerebrospinal fluid and serum GABA concentrations in manic patients, with significant negative correlations between GABA levels and Hamilton Depression Scale (HAM-D) scores (Daniele et al., 2012; Chen et al., 2010). This inhibitory deficit may arise from selective damage to ventral tegmental area GABAergic neurons (Malhi et al., 2013), compounded by neurotrophic factor dysregulation (e.g., reduced BDNF levels) (Chanana and Kumar, 2016). AMPH-induced animal models provide mechanistic insights: acute administration promotes striatal GABA synthesis through postsynaptic 5-HT2C receptor activation (Chanana and Kumar, 2016), whereas chronic exposure downregulates GABA transporter GAT-1 expression, impairing synaptic clearance and triggering GAD-negative feedback inhibition. This results in biphasic GABA alterations (“up-down” trajectory) that may underlie acute-to-chronic pathological progression (Zhang et al., 2022).

Emerging evidence highlights complex GABA-glutamate interactions: manic patients exhibit reduced Glu/GABA ratios, with glutamatergic contributions to this imbalance exceeding GABAergic effects by 2.3-fold (Scotti-Muzzi et al., 2021). Multimodal approaches integrating neuroimaging and electrophysiology will be critical to dissecting this dynamic coupling. While GABA transporter function shows promise as a translational biomarker, diagnostic specificity remains limited by cross-disorder overlap (e.g., anxiety disorders also exhibit GABAergic dysregulation) (Fink and Göthert, 2007).

### 4.4 5-hydroxytryptamine

The serotonergic (5-hydroxytryptamine—5-HT) system plays a pivotal role in the neurobiology of mania. As one of the most widely distributed neurotransmitter systems in the central nervous system (CNS), serotonergic neurons originate primarily from the dorsal raphe nucleus and median raphe nucleus. Their extensive projections innervate key brain regions including the thalamus, hypothalamus, striatum, hippocampus, and prefrontal cortex, regulating higher-order functions such as sleep-wake cycles, emotional processing, and impulse control (Ye et al., 2021). This widespread neuroanatomical architecture provides the structural basis for serotonergic involvement in manic pathophysiology.

Clinical investigations have revealed significant serotonergic dysregulation during manic episodes, characterized by reduced prefrontal and temporal cortical 5-HT concentrations inversely correlated with Young Mania Rating Scale (YMRS) scores (Hedya et al., 2024). Mechanistically, this disturbance arises from bidirectional regulatory dysfunction: Tryptophan hydroxylase 2 (TPH2) activity impairment diminishes 5-HT biosynthesis (Sandoval-Sánchez et al., 2020), while concurrently, elevated monoamine oxidase A (MAOA) activity enhances 5-HT degradation (Stautland et al., 2023). This synthesis-degradation imbalance initiates compensatory feedback mechanisms via 5-HT1A autoreceptor-mediated inhibition of dorsal raphe nucleus neuronal firing, ultimately reducing synaptic cleft 5-HT availability (Bordon, 2022). Notably, receptor subtype-specific alterations manifest as 5-HT1B upregulation coupled with 5-HT1A and 5-HT2A receptor downregulation (Bouchette et al., 2024), suggesting receptor profile remodeling constitutes a critical pathophysiological node. Preclinical validation emerges from AMPH-induced manic models demonstrating biphasic 5-HT fluctuations: Acute phase (24 h) elevation mediated through 5-HT2C receptor-dependent vesicular release, contrasting with chronic phase (7d) depletion driven by MAOA hyperactivity and TPH2 phosphorylation suppression (Chaves-Filho et al., 2024). This dynamic engages GABAergic negative feedback circuitry—5-HT2C activation via PLCβ signaling upregulates Fos expression in GABAergic interneurons, which subsequently inhibit 5-HT neurons through GABAA receptors, forming a self-reinforcing pathological loop of serotonergic suppression and GABAergic hyperactivation (Boothman et al., 2006).

Current therapeutic strategies for mania are intricately linked to serotonergic system modulation. Lithium exerts its effects by inhibiting GSK-3β phosphorylation, thereby augmenting 5-HT1A receptor responsiveness and enhancing synaptic vesicle release. Valproate, conversely, upregulates TPH2 gene expression through histone deacetylase inhibition (Gubert et al., 2020). These mechanistic insights have informed the development of novel agents including 5-HT transporter modulators (e.g., vilazodone) and receptor subtype-specific agonists (e.g., 5-HT1B/1D ligands). Despite these advances, clinical translation faces significant hurdles. Overlapping serotonergic profiles between bipolar disorder and major depressive disorder complicate differential diagnosis, while incomplete characterization of receptor subtype regional specificity limits therapeutic precision (Shiah and Yatham, 2000). Future research should prioritize optogenetic mapping of spatiotemporal 5-HT receptor dynamics, development of positron emission tomography (PET) ligands for *in vivo* receptor subtype quantification, and elucidation of epigenetic mechanisms underlying TPH2 regulation in vulnerable brain regions. These approaches may uncover region-specific serotonergic signatures, enabling the design of circuit-targeted interventions with improved therapeutic indices.

### 4.5 Norepinephrine, NE

Hyperfunction of the norepinephrine (NE) system represents a core pathological mechanism in mania (Underhill et al., 2020). As a primary catecholaminergic neurotransmitter system, noradrenergic neurons originate in brainstem nuclei including the locus coeruleus and raphe nuclei. Their extensive projections innervate key limbic and cortical regions involved in emotional regulation, including the prefrontal cortex, temporal lobe, hippocampus, and amygdala. Through α1/2 and β1/2 adrenergic receptor subtypes, this system mediates critical functions such as emotional arousal, stress responsiveness, and cognitive control (Waples, 2022).

The classical catecholamine hypothesis posits that manic episodes arise from noradrenergic (NE) system hyperactivation, supported by lithium’s therapeutic effects via NE release inhibition and manic patients’ 40-60% increased urinary MHPG excretion (Fountoulakis et al., 2022). Pathophysiology involves: Aberrant locus coeruleus activation triggers ERK-mediated tyrosine hydroxylase (TH) phosphorylation, tripling NE biosynthesis (Zeng et al., 2023). Concurrently, PKC-mediated NE transporter (NET) phosphorylation reduces reuptake efficiency while presynaptic α2 receptor desensitization creates a “release-reuptake” positive feedback loop (Hayley et al., 2021). Chronic adaptive changes include β-receptor downregulation and α1-receptor upregulation, potentially mediating acute-to-chronic symptom transitions. AMPH-induced animal models demonstrate dose-dependent prefrontal NE elevation correlating with stereotypy scores (Underhill et al., 2020). Two convergent mechanisms drive this phenotype: MAO inhibition reduces NE metabolism to MHPG, while HPA axis activation enhances TH transcription via glucocorticoid receptors (Snyder and Innis, 1979). This dual action leads to synaptic NE accumulation, activating amygdala β1 receptors for emotional arousal while impairing prefrontal α2 receptor-mediated cognitive control.

Current therapeutic strategies target multiple components of the noradrenergic system: α2 agonists (e.g., clonidine) attenuate locus coeruleus firing via presynaptic negative feedback, reducing NE release; β-blockers (e.g., propranolol) mitigate postsynaptic hyperactivation by antagonizing β-adrenoceptors. Despite these mechanistic insights, clinical translation remains challenging due to overlapping NE dysregulation in anxiety disorders and post-traumatic stress disorder, combined with incomplete characterization of receptor subtype regional specificity (Dean and Keshavan, 2017). Future research should prioritize integrating PET/MRI neuroimaging to map spatiotemporal expression profiles of NE receptor subtypes, conducting pharmacogenetic studies to identify NET gene polymorphisms associated with treatment response, and developing subtype-selective ligands to dissect receptor-specific contributions to symptom domains. These initiatives may uncover biomarkers for NE-related endophenotypes, enabling the development of precision medicine approaches tailored to individual receptor expression signatures.

### 4.6 Acetylcholine, ACh

The cholinergic system demonstrates multifaceted involvement in the pathophysiology of mania, with acetylcholine (ACh) neurotransmission exhibiting complex regulatory dynamics across neuroanatomical circuits (Yang et al., 2023). As a pivotal neuromodulator, ACh-producing neurons originating from the basal forebrain complex extensively innervate cognition-associated regions including prefrontal and temporal cortices as well as the hippocampus, coordinating higher-order functions through synergistic activation of nicotinic (nAChRs) and muscarinic (mAChRs) receptor subtypes (Cox et al., 2020).

The cholinergic-adrenergic equilibrium hypothesis posits that manic states arise from noradrenergic hyperactivation coupled with cholinergic suppression, a paradigm substantiated by both preclinical models and clinical observations (Chen et al., 2010). AMPH-induced mania model reveal striatal ACh fluctuations characterized by acute-phase (24 h) elevation mediated through D2 receptor-dependent acetylcholinesterase (AChE) inhibition, contrasting with chronic-phase (7d) normalization via protein kinase C (PKC)-mediated suppression of choline acetyltransferase (ChAT) expression (Bashkatova and Philippu, 2019; Varela et al., 2013). This biphasic phenomenon originates from DA-mediated regulation of cholinergic transmission, establishing an adaptive homeostatic loop where acute dopaminergic surges transiently elevate synaptic ACh through AChE blockade, while chronic DA exposure induces sustained cholinergic depletion via transcriptional ChAT downregulation.

Notably, interspecies divergence complicates translational interpretation: whereas rodent models demonstrate acute ACh elevation, manic patients exhibit reduced cerebrospinal ACh levels (Varela et al., 2013). This discrepancy may stem from 40% greater prefrontal mAChR density in humans versus rodents, coupled with M1/M4 subtype-selective downregulation and α1 adrenergic receptor-mediated suppression of ACh release during noradrenergic hyperactivity (Lutfy et al., 2024). Future investigations should prioritize developing computational models of ACh-NE cross-system dynamics across neural networks, particularly elucidating mAChR subtype-specific contributions to emotional regulation—especially M1 receptor-mediated cognitive potentiation effects. Such mechanistic insights will advance understanding of cholinergic pathophysiology in bipolar disorders and inform multi-target therapeutic strategies addressing neurotransmitter network dysregulation (Table 4).

**TABLE 4:** Neurochemical dysregulation profiles in mania: regional pathophysiology and therapeutic targets.

Neuro transmitter system	Core brain regions	Clinical pathological features	Key findings in AMPH Models	Dynamic regulation	Therapeutic relevance
DA	Striatum, prefrontal cortex, nucleus accumbens, hippocampus	Elevated DA levels during mania; D2 receptor hypersensitivity with D1 receptor dysfunction	Acute exposure: striatal DA surge; Chronic exposure: Prefrontal DA metabolic imbalance	Limbic DA hyperactivation; prefrontal DA imbalance	Second-generation antipsychotics
Glu	Hippocampus, striatum, nucleus accumbens	Increased Glu uptake in limbic system; Glutamine level abnormalities	AMPAR/KAR overactivation; EAATs dysfunction	Synaptic Glu accumulation	mGluR-targeted allosteric modulators
GABA	Prefrontal cortex, hippocampus, nucleus accumbens	Reduced CSF GABA; Altered Glu/GABA ratio	Acute AMPH: Striatal GABA↑; Chronic AMPH: GAT-1↓	Biphasic “rise-fall” pattern	Benzodiazepines
5-HT	Raphe nucleus, prefrontal cortex, hippocampus	Prefrontal 5-HT↓, 5-HT1B↑, 5-HT1A/2A↓	Acute AMPH: Hippocampal 5-HT↑; Chronic: MAOA↑-mediated catabolism	Negative feedback loop	Lithium salts
NE	Locus coeruleus, prefrontal cortex, amygdala	Elevated urinary MHPG; β-receptor↓, α_1_ receptor↑	NE metabolism↓; HPA axis activation with TH↑	Positive feedback cycle	α_2_agonists, β-blockers
ACh	Basal forebrain, prefrontal cortex, striatum	CSF ACh↓; M1/M4 receptor↓	Acute: ACh↑ via AChE↓; Chronic: ChAT↓	DA-modulated biphasic shift	Cholinesterase inhibitors

## 5 Molecular mechanisms of mania-associated neural signaling pathways

### 5.1 Neuroplasticity and cell survival related pathways

#### 5.1.1 The ERK signaling pathway: a central regulator of neuroplasticity

The extracellular signal-regulated kinase (ERK) pathway, a pivotal branch of the mitogen-activated protein kinase (MAPK) cascade, orchestrates neurodevelopmental and neuroplastic processes through phosphorylation-dependent regulation of transcription factors and cytoskeletal proteins, critically modulating neurogenesis, synaptic plasticity, and neuronal survival (Park, 2023). Clinical neuropathological analyses reveal reduced total ERK1/2 protein expression in the prefrontal cortices of bipolar manic patients compared to healthy controls, with the degree of reduction inversely correlating with cognitive flexibility deficits—implicating ERK hypofunction in manic-related neuroplasticity impairment (Tan et al., 2023). Mood stabilizers lithium and valproate (VPA) reverse this pathology by enhancing prefrontal ERK1/2 phosphorylation, mechanistically through glycogen synthase kinase-3β (GSK-3β) inhibition-mediated disinhibition of Raf kinase activity (Owens et al., 2012; Walz et al., 2008).

Preclinical validation emerges from AMPH-induced mania model demonstrating hippocampal CA1-specific ERK1 phosphorylation deficits concurrent with dendritic spine density reduction and attenuated long-term potentiation (LTP) magnitude (Valvassori et al., 2019b). Crucially, ERK activity blockade abolishes brain-derived neurotrophic factor (BDNF)-stimulated synaptic protein synthesis, positioning ERK signaling as a central integrator of neurotrophin-mediated synaptic remodeling.

Mechanistically, ERK regulates neuronal adaptability via a three-tiered kinase cascade culminating in immediate-early gene (IEG) induction (e.g., c-Fos, Egr-1), thereby coordinating structural and functional plasticity. AMPH-induced dopamine D1 receptor overactivation may suppress this pathway via aberrant cAMP/protein kinase A (PKA) signaling. Future investigations should employ cell type-specific knockout models to dissect isoform-selective ERK contributions (ERK1 vs. ERK2) to discrete manic symptomatology domains, particularly affective dysregulation versus cognitive disintegration.

#### 5.1.2 The CREB signaling pathway: a hub connecting synaptic plasticity to neurotrophic regulation

The cAMP response element-binding protein (CREB), a master transcriptional integrator of protein kinase A (PKA) and extracellular signal-regulated kinase (ERK) signaling, critically regulates neuronal plasticity and stress adaptation through genomic control of brain-derived neurotrophic factor (BDNF) and B-cell lymphoma 2 (Bcl-2) expression (Yan et al., 2024). Clinical neuroimaging reveals diminished prefrontal cortical CREB phosphorylation in bipolar disorder patients, with phosphorylation deficits inversely correlating with cognitive flexibility impairments, suggesting CREB hypofunction as a molecular substrate for manic-related cognitive dysfunction (Goloncser et al., 2024).

AMPH-induced mania model exhibit pronounced CREB dysregulation: Dorsal striatal CREB phosphorylation declines significantly at 24 h post-administration, paralleling BDNF protein reduction (Cechinel-Recco et al., 2012). Crucially, CREB activity suppression selectively attenuates AMPH-driven hyperlocomotion, directly implicating CREB signaling in manic behavioral pathogenesis. Lithium carbonate demonstrates therapeutic reversibility, normalizing striatal CREB phosphorylation and upregulating synaptophysin via CREB-BDNF transcriptional coupling (Walz et al., 2008).

Mechanistic analyses position CREB as a nodal convergence point for ERK/PKA signaling cascades, governing BDNF-Tropomyosin receptor kinase B (TrkB) pathway activation. AMPH-induced dopamine D1 receptor hyperactivation triggers pathological cAMP/PKA signaling that suppresses CREB phosphorylation, ultimately impairing synaptic plasticity through BDNF synthesis deficits.

### 5.2 Inflammation and immunoregulatory pathways

#### 5.2.1 The COX-2-IDO-1 pathway: the crossroads of neuroinflammation and glutamatergic disorders

The cyclooxygenase-2 (COX-2)-indoleamine 2,3-dioxygenase 1 (IDO-1) axis critically modulates neuroimmune homeostasis through coordinated regulation of prostaglandin biosynthesis and tryptophan catabolism. Clinical biomarker analyses demonstrate elevated serum COX-2 activity in bipolar manic patients compared to healthy controls, suggesting pathway hyperactivation may disrupt excitatory-inhibitory balance via dual glutamatergic mechanisms (Zhang et al., 2023). Firstly, COX-2-driven astrocytic endocytosis impairs synaptic glutamate reuptake efficiency. Secondly, IDO-1-mediated tryptophan-kynurenine pathway shunting causes quinolinic acid (QUIN) accumulation, which potentiates N-methyl-D-aspartate receptor (NMDAR)-dependent glutamatergic hyperexcitability (Tran et al., 2020). Therapeutic co-administration of meloxicam (COX-2 inhibitor) and 1-methyl-DL-tryptophan (IDO-1 inhibitor) normalizes striatal glutamate levels, validating this pathway’s translational potential (Valvassori et al., 2019b).

AMPH-induced mania model precisely recapitulate this neuroinflammatory signature, with striatal COX-2 overexpression and IDO-1 activation emerging within 6 h post-administration. Mechanistically, the COX-2-IDO-1 cascade engages oxidative stress through QUIN-induced mitochondrial reactive oxygen species (ROS) overproduction, which activates NLRP3 inflammasomes to amplify interleukin-1β (IL-1β) secretion and COX-2 expression—establishing a self-reinforcing inflammatory loop.

Future investigations should delineate cell type-specific activation patterns (glia vs. neurons) and characterize spatiotemporal correlations between peripheral inflammation markers and central COX-2-IDO-1 activity dynamics.

#### 5.2.2 The P2 × 7 receptor pathway: neuroimmune crosstalk in bipolar MANIA

The P2 × 7 receptor (P2 × 7R), an ATP-gated cation channel, orchestrates neuroinflammatory responses through NLRP3 inflammasome activation, serving as a critical mediator of neuroimmune interactions in bipolar disorder (Yu et al., 2023). Clinical multi-omics analyses demonstrate elevated P2 × 7R mRNA expression in peripheral blood mononuclear cells (PBMCs) of manic-phase patients compared to euthymic controls, with expression levels positively correlating with serum interleukin-1β (IL-1β) concentrations—identifying this pathway as a putative biomarker for manic-associated neuroinflammation (Gubert et al., 2016).

Preclinical validation emerges from AMPH-induced mania model, where striatal P2 × 7R protein upregulation emerges within 3 h post-AMPH administration, paralleling extracellular ATP accumulation. This neuroinflammatory signature is mechanistically attributed to ATP-P2 × 7R-NLRP3 signaling axis activation (Iwata et al., 2016).

Pathway integration analyses reveal P2 × 7R signaling synergizes with the COX-2-IDO-1 cascade to form an inflammatory amplification network: IL-1β initiates cyclooxygenase-2 (COX-2)-dependent prostaglandin E2 (PGE2) synthesis, which subsequently enhances ATP release through pannexin-1 channels, establishing a self-reinforcing feedforward circuit (“IL-1β→COX-2→PGE2→ATP”). This crosstalk potentially drives glutamatergic hyperexcitability and dopaminergic dysregulation via NMDA receptor potentiation and striatal terminal activation.

### 5.3 Metabolic and energy homeostasis pathways

#### 5.3.1 The GLP-1 receptor pathway: metabolic adaptation and cross-system interactions in mania

The glucagon-like peptide-1 receptor (GLP-1R) signaling pathway, a critical neuromodulatory system governing cognitive and metabolic homeostasis (Zhang et al., 2023), exhibits putative hyperactivation in bipolar mania. While clinical evidence remains limited, preclinical models suggest compensatory GLP-1R pathway engagement during manic states through coordinated molecular adaptations.

In AMPH-induced mania model, GLP-1R signaling displays a biphasic activation profile characterized by Gαs-protein-mediated adenylate cyclase potentiation and subsequent Akt pathway activation via phosphoinositide 3-kinase (PI3K)-dependent cascades (Chaves Filho et al., 2020). This dual signaling axis induces widespread phosphorylation of Akt substrates, forming a neuroprotective network to counteract AMPH-driven mitochondrial dysfunction and oxidative stress. Mechanistically, pathway hyperactivity arises from synergistic inputs: (1) neurotransmitter imbalance-induced metabolic crisis, (2) oxidative stress-triggered mitochondrial depolarization, and (3) direct AMPH activation of aberrant cAMP-protein kinase A (PKA) signaling (de Souza Gomes et al., 2015).

Notably, GLP-1R functions within an integrated signaling framework where it synergizes with the cAMP/PKA axis to drive transcriptional reprograming and cellular resilience while modulating monoaminergic tone through D2 receptor phosphorylation and glutamate transporter (EAAT) regulation. Future research should prioritize clinical validation using GLP-1R PET ligands to quantify receptor density in manic versus euthymic states, development of brain-region-specific knockout models to dissect circuit-level contributions, and pharmacological interrogation of the GLP-1R-PKA-Akt axis as a potential anti-manic target.

### 5.4 Enzymes and epigenetic pathways

#### 5.4.1 The HDAC pathway: epigenetic dysregulation in mania

The histone deacetylase (HDAC) signaling pathway contributes to manic pathophysiology through chromatin remodeling and transcriptional regulation (Ho et al., 2020). HDAC hyperactivity promotes histone deacetylation, leading to chromatin condensation and repression of inhibitory neuronal genes while HDAC inhibitors like valproate enhance neurotrophic factor expression (BDNF, NGF) and synaptic plasticity. Clinical evidence supports HDAC overactivation in bipolar disorder, with valproate’s therapeutic efficacy directly linking HDAC inhibition to symptom remission (Varela et al., 2020).

In AMPH-induced mania model, frontal cortex HDAC elevation occurs alongside mitochondrial respiratory chain dysfunction, suggesting energy deficits trigger HDAC-mediated adaptive gene expression (Steckert et al., 2013; Moretti et al., 2011). Concurrent oxidative stress may further activate HDACs, creating a feedforward loop of transcriptional dysregulation.

HDAC signaling intersects with DNMT activity to synergistically regulate chromatin states, modulates dopamine D2 receptor expression in reward circuitry, and interacts with lithium’s GSK-3β inhibitory effects. While HDAC’s therapeutic actions depend on DNMT interactions, the precise regulatory network remains unclear (Stertz et al., 2014). Future multi-omic studies mapping HDAC-DNMT crosstalk in vulnerable brain regions and validating HDAC isoform-specific inhibitors hold translational potential.

### 5.5 Ion channel

#### 5.5.1 The Kv7 (KCNQ) signaling pathway: voltage-gated potassium channels in mania

The Kv7 (KCNQ) voltage-gated potassium channel family modulates neuronal excitability and contributes to manic pathophysiology (Mondejar-Parreño et al., 2020). While preclinical studies demonstrate therapeutic potential, clinical translation remains underexplored. Non-selective Kv7 activators like retigabine exert antimanic effects in animal models by targeting multiple channel subtypes, validating this pathway as a drug discovery target (Grunnet et al., 2014).

In AMPH-induced mania model: Kv7 activation reduces striatal dopamine release through dual mechanisms: hyperpolarizing VTA dopamine neuron resting membrane potential via enhanced potassium efflux and attenuating burst firing dynamics by stabilizing action potential thresholds (Kristensen et al., 2012). These effects collectively decrease synaptic dopamine concentrations and mitigate reward-related behaviors.

Notably, antimanic agents like lithium synergize with Kv7 activation through GSK-3β inhibition, suggesting convergent pathways in reducing neuronal hyperexcitability (Mondejar-Parreño et al., 2020). However, subtype-specific challenges persist: Kv7.2/Kv7.3 subunits predominate in brain regions relevant to mood regulation, while peripheral expression of other isoforms (e.g., Kv7.1) may cause side effects. Development of brain-penetrant, subtype-selective ligands will be critical for translational success. Future studies should prioritize *in vivo* Kv7 channel imaging in manic patients, isoform-specific knockout models to dissect circuit contributions, and structure-based drug design targeting Kv7.2/Kv7.3 gating mechanisms. This integrative approach may unlock Kv7 channels as novel therapeutic targets for bipolar disorder.

### 5.6 The PKCβ-DAT pathway: neurotransmitter-receptor interaction pathway

Protein kinase C (PKC) signaling plays a central role in the pathophysiology and treatment of bipolar disorder (Fallah et al., 2016). Postmortem studies demonstrate elevated PKC activity in the prefrontal cortex of manic patients compared to healthy controls, with significant positive correlations between PKC activation and dopamine transporter (DAT) phosphorylation levels (Zarate et al., 2007; Goloncser et al., 2024). This suggests PKC hyperactivity drives dopaminergic system dysfunction through DAT regulation.

In AMPH-induced mania model, TAAR1 receptor activation in the ventral tegmental area (VTA) initiates a signaling cascade involving Gαi inhibition, cAMP elevation, and calcium influx (Goloncser et al., 2024; Trevizol et al., 2011). This second messenger surge activates PKCβ via calmodulin-dependent pathways, leading to N-terminal serine phosphorylation of DAT. Conformational changes in DAT promote dopamine efflux, concurrent with increased striatal COX-2 expression and 8-isoprostane F2α accumulation—hallmarks of oxidative stress (Valvassori et al., 2019a). This creates a self-reinforcing cycle where dopamine efflux enhances oxidative stress, activating phospholipase C (PLC) and perpetuating PKCβ activation. Pharmacological targeting of this pathway shows promise. The selective PKCβ inhibitor lubeluzole restores dopaminergic homeostasis by blocking DAT phosphorylation and upregulating D2 receptor membrane expression (Zestos et al., 2019). Mechanistically, lubeluzole reduces 4-hydroxynonenal (HNE) accumulation and attenuates JNK/c-Jun-mediated neuronal apoptosis (Kawano et al., 2021).

However, PKCβ-DAT signaling operates within a broader network: inflammatory feedback with COX-2/PGE2 amplifies neuroinflammation, ERK/CREB pathway suppression impairs neuroplasticity, and concurrent 5-HT/NE transporter phosphorylation disrupts monoamine balance. This interconnected signaling network suggests that PKCβ represents a critical therapeutic node. Combining PKCβ inhibition with antioxidant/anti-inflammatory strategies may break pathological cycles and achieve precision intervention in mania (Dencker et al., 2008). Future studies should prioritize *in vivo* PKCβ activity imaging in bipolar patients, development of brain-penetrant isoform-selective PKC modulators, and systems biology approaches to map PKCβ interactomes in vulnerable circuits. Such integrative strategies could transform PKC signaling from a mechanistic target to a clinical reality (Table 5).

**TABLE 5:** Molecular mechanisms of mania-associated neural signaling pathways.

Pathway	Molecular mechanism	Clinical evidence	Preclinical validation	Therapeutic implications	Future directions
ERK signaling	- MAPK cascade regulating neuroplasticity via transcription factor phosphorylation	- Reduced prefrontal ERK1/2 in manic patients; inverse correlation with cognition	- AMPH models: hippocampal ERK1↓, dendritic spine density↓, LTP↓	- Lithium/VPA enhance ERK phosphorylation via GSK-3β inhibition	- ERK isoform-specific roles using conditional knockouts
CREB signaling	- Integrates PKA/ERK to regulate BDNF/Bcl-2 transcription	- Prefrontal CREB phosphorylation↓ correlates with cognitive deficits	- AMPH models: striatal CREB↓ at 24h; BDNF↓; CREB inhibition blocks hyperactivity	- Lithium restores CREB phosphorylation via BDNF axis	- Mechanistic links between cAMP/PKA dysregulation and CREB suppression
COX-2-IDO-1 axis	- Dual modulation: COX-2↑ impairs Glu reuptake; IDO-1↑ generates QUIN→NMDAR activation	- Serum COX-2↑ in mania; QUIN↑ correlates with neuroinflammation	- AMPH models: striatal COX-2↑/IDO-1↑ at 6h; QUIN→ROS→NLRP3 inflammasome activation	- COX-2/IDO-1 inhibitors normalize striatal Glu	- Glia-neuron activation profiling; peripheral-central inflammation dynamics
P2 × 7R pathway	- ATP-P2 × 7R-NLRP3 axis drives IL-1β release; synergizes with COX-2-IDO-1	- PBMC P2 × 7R mRNA↑ in mania; correlates with IL-1β↑	- AMPH models: striatal P2 × 7R↑ at 3h; ATP↑→inflammatory loop	- P2 × 7R antagonists reduce Glu/Dopamine dysregulation	- Brain-specific P2 × 7R modulators; spatiotemporal mapping of neuroinflammation
GLP-1R pathway	- Gαs→adenylate cyclase→PI3K/Akt activation; counteracts oxidative stress	- Limited clinical data; putative compensatory activation in mania	- AMPH models: biphasic Akt activation; mitigates mitochondrial dysfunction	- GLP-1R agonists may stabilize metabolic-cognitive networks	- GLP-1R PET imaging; isoform-specific CRISPR models; Akt/PKA inhibitors
HDAC signaling	- Chromatin REMODELING via histone deacetylation; represses neuroprotective genes	- HDAC↑ in bipolar patients; valproate efficacy linked to HDAC inhibition	- AMPH models: frontal HDAC↑ with mitochondrial dysfunction	- HDAC inhibitors (e.g., valproate) enhance BDNF expression	- HDAC-DNMT crosstalk mapping; isoform-specific inhibitors
Kv7 channels	- K + efflux↓ neuronal excitability; stabilizes membrane potential	- Underexplored clinically; retigabine shows preclinical efficacy	- AMPH models: Kv7 activation↓ striatal DA via VTA hyperpolarization	- Kv7 activators (retigabine) reduce DA release; synergize with lithium	- Subtype-selective Kv7.2/7.3 ligands; *in vivo* Kv7 imaging in patients
PKCβ-DAT pathway	- PKCβ phosphorylates DAT→DA efflux↑; oxidative stress→PLC/PKCβ feedback	- Prefrontal PKC↑ correlates with DAT phosphorylation↑ in mania	- AMPH models: TAAR1→PKCβ→DAT phosphorylation→DA↑; oxidative markers↑	- PKCβ inhibitors (lubeluzole) restore DA homeostasis; reduce apoptosis	- *In vivo* PKCβ imaging; isoform-specific modulators; systems biology approaches

## 6 Mechanism of oxidative stress-inflammation axis in AMPH-induced mania

### 6.1 Elevated levels of oxidative stress

Oxidative stress, characterized by disrupted equilibrium between reactive oxygen species (ROS) production and antioxidant defenses, induces macromolecular damage through lipid peroxidation, protein modification, and DNA oxidation—mechanisms implicated in numerous neuropsychiatric disorders (Sies, 2015). Clinical investigations reveal heightened oxidative burden in bipolar manic patients, evidenced by elevated peripheral and cerebral malondialdehyde (MDA) levels and protein carbonyl content, coupled with diminished antioxidant enzyme activities including superoxide dismutase (SOD) and glutathione peroxidase (GPx) (Martino et al., 2016). The underlying pathophysiology involves dopamine (DA) metabolic dysregulation: Mania-associated dopaminergic hyperactivity drives excessive DA catabolism via monoamine oxidase (MAO)-mediated hydrogen peroxide (H_2_O_2_) generation, which undergoes Fenton reactions with iron to yield neurotoxic 6-hydroxydopamine (6-OHDA). Concurrent DA autoxidation produces dopaminergic quinones and ROS, triggering mitochondrial electron transport chain dysfunction, antioxidant enzyme suppression, and neuroinflammatory cascades. These interconnected mechanisms establish a self-perpetuating “oxidative injury-neuronal degeneration-symptom exacerbation” cycle (He et al., 2023).

**FIGURE 1:**
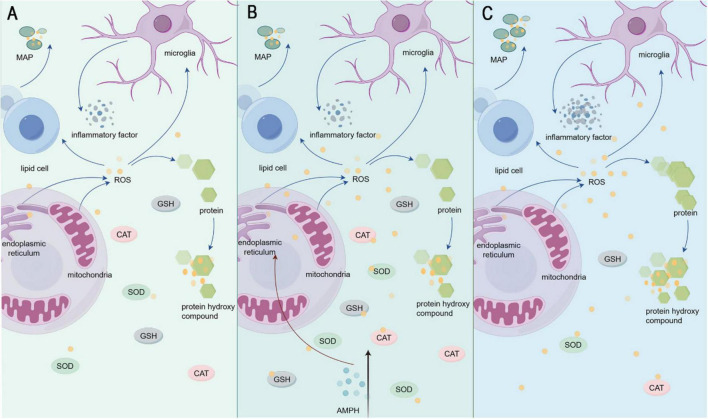
**(A)** Shows the state of inflammatory factors and oxidative stress factors under normal conditions in the organism. **(B)** Indicates AMPH injection. **(C)** Shows the change of inflammatory factors and oxidative stress factors in the organism after AMPH injection.

The AMPH-induced mania model faithfully recapitulates clinical oxidative stress dynamics (Figure 1), with acute exposure triggering marked elevation of malondialdehyde (MDA) and protein carbonyls in rat prefrontal cortex, hippocampus, and striatum (Frey et al., 2006b; Valvassori et al., 2011). Chronic AMPH administration amplifies oxidative damage through histone deacetylase (HDAC)-mediated upregulation of 4-hydroxynonenal (4-HNE), 8-iso-prostaglandin F2α (8-iso-PGF2α), and 3-nitrotyrosine (3-NT) (Menegas et al., 2019). Notably, AMPH exerts tissue-specific oxidative effects: sustained cerebral lipid peroxidation contrasts with reduced serum thiobarbituric acid reactive substances (TBARS), likely reflecting peripheral antioxidant compensation via Entpd3-driven transient GPx/SOD upregulation (Dencker et al., 2008; Valvassori et al., 2019a). Mechanistically, AMPH disrupts mitochondrial bioenergetics by inhibiting tricarboxylic acid (TCA) cycle enzymes (e.g., α-ketoglutarate dehydrogenase) while upregulating indoleamine 2,3-dioxygenase-1 (IDO-1), synergistically driving reactive oxygen species (ROS) overproduction and antioxidant system collapse (Tran et al., 2020; Trevizol et al., 2011).

Modulating oxidative stress presents innovative therapeutic avenues for bipolar disorder management. Emerging strategies leverage oxidative damage biomarkers such as cerebrospinal fluid malondialdehyde (MDA) and serum 8-iso-prostaglandin F2α (8-iso-PGF2α) as objective indicators for disease activity staging and treatment response monitoring (Giménez-Palomo et al., 2024). Mechanistic approaches involve suppressing indoleamine 2,3-dioxygenase-1 (IDO-1) activity to attenuate kynurenine pathway-derived radical generation (Menegas et al., 2020), administering tricarboxylic acid (TCA) cycle modulators like α-lipoic acid to restore mitochondrial bioenergetics (Steckert et al., 2013), and combining lithium with N-acetylcysteine (NAC) to synergistically enhance glutathione synthesis while detoxifying dopamine quinones (Menegas et al., 2019). Novel therapeutic development focuses on HDAC inhibitors and GLP-1 receptor agonists that concurrently regulate epigenetic modifications and redox homeostasis, enabling multi-target intervention (Giménez-Palomo et al., 2024). Future research should prioritize multidisciplinary investigations integrating oxidative stress profiling with neuroinflammatory and epigenetic biomarkers, coupled with stratified clinical trials to validate precision therapeutic protocols.

### 6.2 Decreased antioxidant capacity due to decreased GSH levels

Glutathione (GSH), a tripeptide comprising glutamate, cysteine, and glycine, serves as a master regulator of intracellular redox homeostasis through reactive oxygen species (ROS) scavenging and xenobiotic detoxification (Lima et al., 2022). Clinical studies demonstrate significant GSH depletion in blood and cerebral tissues of manic patients, correlating with oxidative stress-induced metabolic dysregulation (Yap et al., 2010). Mechanistically, this imbalance arises from dual pathways: accelerated GSH catabolism via glutathione peroxidase (GPx) and glutathione S-transferase (GST) hyperactivity, coupled with impaired synthesis due to inflammatory cytokine-mediated (IL-6, TNF-α) suppression of glutamate-cysteine ligase (GCL) and mitochondrial dysfunction (Averill-Bates, 2023; Jiménez-Fernández et al., 2021).

The AMPH-induced mania model recapitulates these findings, showing pronounced GSH depletion in prefrontal, hippocampal, and amygdalar regions (de Souza Gomes et al., 2015). AMPH-driven dopamine release potentiates ROS generation (superoxide anions, hydroxyl radicals) during oxidative metabolism, triggering lipid peroxidation (elevated MDA) and exhausting GSH reserves to mitigate oxidative damage (Valvassori et al., 2013). Notably, GLP-1 receptor agonists (e.g., liraglutide) and lithium restore GSH levels in this model, revealing functional crosstalk between GSH metabolism, neurotrophic signaling, and classical mood stabilization pathways (Chaves Filho et al., 2020).

Emerging therapeutic strategies targeting GSH restoration show translational promise. Supplementation with GSH precursors like N-acetylcysteine enhances endogenous antioxidant capacity, effectively ameliorating manic-like behaviors in preclinical models. Pharmacological activation of the Nrf2 pathway offers another viable approach to boost GSH synthesis. The combined use of traditional mood stabilizers (e.g., lithium) with antioxidants demonstrates synergistic effects in redox homeostasis restoration, while novel GLP-1R agonists show potential for multi-target intervention by bridging metabolic and neurotrophic regulation. Future investigations should prioritize clinical validation of these GSH-modulating approaches and evaluate peripheral oxidative stress biomarkers (e.g., GSH/MDA ratio) as objective metrics for treatment efficacy assessment.

### 6.3 Elevated levels of body inflammation due to rise in pro-inflammatory factors

The inflammatory response serves as a critical defense mechanism through which the immune system identifies and eliminates pathogens while coordinating tissue repair via immune cell recruitment and adaptive response modulation (Bulut et al., 2021). However, chronic neuroinflammation contributes to psychiatric pathogenesis through proinflammatory cytokine-mediated neurotoxicity. Clinical investigations demonstrate elevated serum and cerebrospinal fluid levels of interleukin-6 (IL-6), tumor necrosis factor-α (TNF-α), and C-reactive protein (CRP) in manic patients, reflecting systemic inflammatory activation (Miola et al., 2022). This persistent inflammation originates from multifactorial mechanisms including chronic psychological stress-induced hypothalamic-pituitary-adrenal (HPA) axis dysregulation, which promotes glucocorticoid resistance and aberrant NF-κB pathway activation (Pereira et al., 2021); mood fluctuation-driven mitochondrial dysfunction that releases damage-associated molecular patterns (DAMPs) to activate microglial TLR4 receptors and inflammatory cascades (Pereira et al., 2021); and blood-brain barrier compromise facilitating peripheral monocyte infiltration into the central nervous system, establishing a bidirectional peripheral-central inflammatory axis (Pereira et al., 2021).

The AMPH-induced mania model accurately replicates clinical neuroinflammatory dynamics, with acute exposure upregulating IL-6 and TNF-α levels in rat prefrontal cortex, hippocampus, and striatum while inducing microglial polarization toward proinflammatory M1 phenotypes (de Souza Gomes et al., 2015; Menegas et al., 2020). Mechanistic studies reveal AMPH’s synergistic activation of inflammatory pathways: dopamine metabolism-derived reactive oxygen species (ROS) oxidatively modify IκBα to activate NF-κB signaling and enhance IL-4/IL-6 transcription (Sakrajda and Szczepankiewicz, 2021); reactive nitrogen species (RNS) induce DNA damage that activates PARP-1/JAK-STAT pathways, upregulating cyclooxygenase-2 (COX-2) and indoleamine 2,3-dioxygenase-1 (IDO-1) (Phan et al., 2020); and mitochondrial ROS leakage triggers NLRP3 inflammasome assembly, driving IL-1β maturation and establishing an oxidative stress-neuroinflammation feedback loop (Valvassori et al., 2015). Notably, AMPH exhibits spatiotemporal specificity in inflammatory modulation, with acute-phase localized brain inflammation (e.g., prefrontal COX-2 elevation) transitioning to chronic-phase peripheral immune infiltration accompanied by compensatory serum IL-10 upregulation (Dencker et al., 2008).

Targeting inflammatory pathways presents novel therapeutic opportunities for bipolar disorder management. Emerging strategies include utilizing inflammatory biomarkers such as cerebrospinal fluid IL-6 and serum CRP levels to guide personalized anti-inflammatory treatment regimens (Korkmaz and Kızgın, 2023). Mechanistically informed interventions encompass small molecule inhibitors (e.g., tofacitinib) to suppress proinflammatory signaling through NF-κB and JAK-STAT pathways, microglial polarization modulators like minocycline to inhibit M1 phenotypic switching, and combination therapies pairing COX-2 inhibitors (celecoxib) with lithium to synergistically reduce IL-6/TNF-α production. Antioxidants such as N-acetylcysteine (NAC) demonstrate adjunctive potential by scavenging reactive oxygen species (ROS) to indirectly inhibit NF-κB activation, with preclinical studies confirming their anti-inflammatory and neuroprotective efficacy. Future investigations should prioritize elucidating peripheral-central inflammatory crosstalk mechanisms while advancing the development of blood-brain barrier-penetrant anti-inflammatory agents for targeted neuromodulation.

## 7 Mechanisms of neurotrophic factors in mania

Neurotrophic factors, encompassing brain-derived neurotrophic factor (BDNF), nerve growth factor (NGF), neurotrophin-3 (NT-3), neurotrophin-4/5 (NT-4/5), and glial cell line-derived neurotrophic factor (GDNF), constitute critical regulators of neuronal survival and plasticity (Gorgulu et al., 2021). Clinical studies reveal distinct neurotrophic alterations in bipolar mania, characterized by reduced BDNF, NGF, and GDNF levels alongside elevated NT-3 and NT-4/5 concentrations—imbalances potentially driving manic behavioral phenotypes (Amodeo et al., 2017; Hodes et al., 2018).

Preclinical investigations using AMPH-induced mania model recapitulate these perturbations: BDNF, NGF, and GDNF expression decreases in the frontal cortex, hippocampus, striatum, and amygdala, while NT-3 and NT-4/5 levels rise in serum and hippocampal tissues (Chaves Filho et al., 2020; Valvassori et al., 2019c; Walz et al., 2008). Mood stabilizers lithium carbonate and valproate normalize these neurotrophic profiles, correlating with attenuated manic-like behaviors (Frey et al., 2006a). Mechanistically, AMPH-induced dopaminergic hyperactivity may upregulate BDNF through D2 receptor-mediated activation of glycogen synthase kinase-3 (GSK-3α/β) and protein kinase C (PKC) signaling cascades (Cechinel-Recco et al., 2012; Valvassori et al., 2011, 2015), while mechanistic underpinnings of other neurotrophic alterations remain undetermined, warranting systematic investigation.

These neurotrophic disruptions likely contribute to neuronal injury and cognitive impairment via impaired synaptic maintenance and neurogenesis. Future therapeutic strategies should prioritize multi-target approaches to normalize neurotrophic homeostasis, potentially through pharmacological modulation of BDNF-TrkB signaling or GDNF receptor tyrosine kinase (RET) activation.

## 8 Challenges and perspectives: translational medicine research directions for the AMPH-induced mania model

The AMPH-induced mania model has emerged as a pivotal tool in bipolar disorder research, enabling comprehensive investigations across behavioral, neurochemical, signaling pathway, oxidative stress, and neuroinflammatory domains. While demonstrating strong face validity in replicating core manic symptoms and neuropathological features, several critical limitations necessitate methodological refinement. Substantial heterogeneity exists in current protocols, particularly regarding dosing parameters (e.g., 2.5-8 mg/kg dose ranges, acute vs. chronic administration schedules) and behavioral quantification methods (e.g., variable Open Field Test metrics), with Table 1 documenting 30% inter-strain pharmacokinetic variability in rodents and Table 2 revealing inconsistent temporal definitions for acute/chronic modeling phases. Protocol standardization should prioritize three key advancements: optimization of strain-specific dose-response curves, implementation of multimodal behavioral assessment systems integrating open field and EPM paradigms, and synergistic application of neuroimaging (fMRI/PET) with region-specific microdialysis to enhance construct validity and reproducibility (Brandon et al., 2003; Chaves-Filho et al., 2024).

Mechanistic understanding remains constrained by oversimplified drug-induced paradigms that neglect gene-environment interplay—a hallmark of bipolar disorder’s multifactorial etiology (Tondo et al., 2017). Advanced modeling strategies should incorporate CRISPR-engineered DAT/BDNF variants to elucidate genetic modulation of AMPH-induced dopaminergic hyperactivity, combine chronic unpredictable mild stress (CUMS) with AMPH exposure to mimic clinical stress-triggered manic transitions (assessed via HPA axis markers and HDAC/DNMT epigenetic profiling), and employ multi-omic approaches to decode AMPH-responsive inflammatory networks like the COX-2-IDO-1 axis (Giménez-Palomo et al., 2024; Pereira et al., 2021; Varela et al., 2024).

Translational innovation requires biomarker-driven therapeutic strategies, including clinical staging systems based on peripheral oxidative stress markers (GSH/MDA ratios) and central inflammatory profiles (CSF IL-6/TNF-α levels), novel drug combinations like GLP-1R agonists with HDAC inhibitors, and patient-specific iPSC-derived neuronal models to map individual differences in AMPH-induced mitochondrial dysfunction (Chaves Filho et al., 2020; Li et al., 2023; Menegas et al., 2020). Through rigorous protocol standardization, multidimensional mechanism elucidation, and precision medicine integration, the AMPH model will remain indispensable for advancing bipolar disorder pathobiology and therapeutic discovery.

## References

[B1] Ackermann T. F. Kempe D. S. Lang F. Lang U. E. (2010). Hyperactivity and enhanced curiosity of mice expressing PKB/SGK-resistant glycogen synthase kinase-3 (GSK-3). *Cell. Physiol. Biochem. Int. J. Exp. Cell. Physiol. Biochem. Pharmacol.* 25 775–786. 10.1159/000315097 20511724

[B2] Ahern T. H. Modi M. E. Burkett J. P. Young L. J. (2009). Evaluation of two automated metrics for analyzing partner preference tests. *J. Neurosci. Methods* 182, 180–188. 19539647 10.1016/j.jneumeth.2009.06.010 PMC2747247

[B3] Amodeo D. A. Grospe G. Zang H. Dwivedi Y. Ragozzino M. E. (2017). Cognitive flexibility impairment and reduced frontal cortex BDNF expression in the ouabain model of mania. *Neuroscience* 345 229–242. 10.1016/j.neuroscience.2016.05.058 27267245 PMC5136525

[B4] Arakawa H. (2023). Revisiting sociability: Factors facilitating approach and avoidance during the three-chamber test. *Physiol. Behav.* 272:114373. 10.1016/j.physbeh.2023.114373 37805136

[B5] Averill-Bates D. A. (2023). The antioxidant glutathione. *Vitamins Hormones* 121 109–141. 10.1016/bs.vh.2022.09.002 36707132

[B6] Bari A. Robbins T. W. (2013). Inhibition and impulsivity: Behavioral and neural basis of response control. *Progr. Neurobiol.* 108 44–79. 10.1016/j.pneurobio.2013.06.005 23856628

[B7] Bartholomew R. E. (1994). Tarantism, dancing mania and demonopathy: The anthro-political aspects of ‘mass psychogenic illness’. *Psychol. Med.* 24 281–306. 10.1017/s0033291700027288 8084927

[B8] Bashkatova V. Philippu A. (2019). Role of nitric oxide in psychostimulant-induced neurotoxicity. *AIMS Neurosci.* 6 191–203. 10.3934/Neuroscience.2019.3.191 32341976 PMC7179361

[B9] Beentjes T. A. A. Goossens P. J. J. Poslawsky I. E. (2012). Caregiver burden in bipolar hypomania and mania: A systematic review. *Perspect. Psychiatric Care* 48 187–197. 10.1111/j.1744-6163.2012.00328.x 23005586

[B10] Bogdanova O. V. Kanekar S. D’Anci K. E. Renshaw P. F. (2013). Factors influencing behavior in the forced swim test. *Physiol. Behav.* 118 227–239. 10.1016/j.physbeh.2013.05.012 23685235 PMC5609482

[B11] Boothman L. Raley J. Denk F. Hirani E. Sharp T. (2006). In vivo evidence that 5-HT(2C) receptors inhibit 5-HT neuronal activity via a GABAergic mechanism. *Br. J. Pharmacol.* 149 861–869. 10.1038/sj.bjp.0706935 17043669 PMC2014685

[B12] Bordon Y. (2022). Platelet-derived 5-HIAA helps neutrophils enter tissue. *Nat. Rev. Immunol.* 22 206–207. 10.1038/s41577-022-00699-z 35194171

[B13] Bouchette D. Fariba K. A. Patel P. Marwaha R. (eds). (2024). “Ziprasidone,” in *StatPearls* (Florida: StatPearls Publishing). 28846230

[B14] Brandon C. L. Marinelli M. White F. J. (2003). Adolescent exposure to methylphenidate alters the activity of rat midbrain dopamine neurons. *Biol. Psychiatry* 54 1338–1344. 10.1016/s0006-3223(03)00787-x 14675797

[B15] Bulut N. Yorguner N. Çarkaxhiu Bulut G. (2021). The severity of inflammation in major neuropsychiatric disorders: Comparison of neutrophil-lymphocyte and platelet-lymphocyte ratios between schizophrenia, bipolar mania, bipolar depression, major depressive disorder, and obsessive compulsive disorder. *Nordic J. Psychiatry* 75:1919201. 10.1080/08039488.2021.1919201 34319861

[B16] Carvalho Pinheiro R. M. Martins, de Lima M. N. Fries G. R. Garcia V. A. Presti-Torres J. (2012). Early life stress exacerbates cognitive dysfunction induced by D-amphetamine: Amelioration by valproic acid. *J. Neural Transmission* 119 627–637. 10.1007/s00702-011-0754-y 22218930

[B17] Cechinel-Recco K. Valvassori S. S. Varela R. B. Resende W. R. Arent C. O. Vitto M. F. (2012). Lithium and tamoxifen modulate cellular plasticity cascades in animal model of mania. *J. Psychopharmacol.* 26 1594–1604. 10.1177/0269881112463124 23076832

[B18] Chanana P. Kumar A. (2016). GABA-BZD receptor modulating mechanism of panax quinquefolius against 72-h sleep deprivation induced anxiety like behavior: Possible roles of oxidative stress, mitochondrial dysfunction and neuroinflammation. *Front. Neurosci.* 10:84. 10.3389/fnins.2016.00084 27013946 PMC4779932

[B19] Chaves Filho A. J. M. Cunha N. L. De Souza A. G. Soares M. V.-R. Jucá P. M. De Queiroz T. (2020). The GLP-1 receptor agonist liraglutide reverses mania-like alterations and memory deficits induced by D-amphetamine and augments lithium effects in mice: Relevance for bipolar disorder. *Prog. Neuro Psychopharmacol. Biol. Psychiatry* 99:109872. 10.1016/j.pnpbp.2020.109872 31954756

[B20] Chaves V. C. Soares M. S. P. Spohr L. Teixeira F. Vieira A. Constantino L. S. (2020). Blackberry extract improves behavioral and neurochemical dysfunctions in a ketamine-induced rat model of mania. *Neurosci. Lett.* 714:134566. 10.1016/j.neulet.2019.134566 31698027

[B21] Chaves-Filho A. J. M. Soares M. V.-R. Jucá P. M. Oliveira T. Q. Clemente D. C. S. Monteiro C. E. S. (2024). Doxycycline reversal of amphetamine-induced mania-like behavior is related to adjusting brain monoamine abnormalities and antioxidant effects in primary hippocampal neurons. *Naunyn-Schmiedeberg’s Arch. Pharmacol.* 397 6017–6035. 10.1007/s00210-024-03009-7 38386042

[B22] Chen J. Fang Y. Kemp D. E. Calabrese J. R. Gao K. (2010). Switching to hypomania and mania: Differential neurochemical, neuropsychological, and pharmacologic triggers and their mechanisms. *Curr. Psychiatry Rep.* 12 512–521. 10.1007/s11920-010-0157-z 20878507

[B23] Chestnykh D. Graßl F. Pfeifer C. Dülk J. Ebner C. Walters M. (2023). Behavioural effects of APH199, a selective dopamine D4 receptor agonist, in animal models. *Psychopharmacology* 240 1011–1031. 10.1007/s00213-023-06347-1 36854793 PMC10006056

[B24] Cho W.-H. Noh K. Lee B. H. Barcelon E. Jun S. B. Park H. Y. (2022). Hippocampal astrocytes modulate anxiety-like behavior. *Nat. Commun.* 13:6536. 10.1038/s41467-022-34201-z 36344520 PMC9640657

[B25] Çiçekli M. N. Tiryaki E. S. Altun A. Günaydın C. (2022). GLP-1 agonist liraglutide improves ouabain-induced mania and depressive state via GSK-3β pathway. *J. Receptor Signal Transduction Res.* 42 486–494. 10.1080/10799893.2022.2032747 35133924

[B26] Clemente A. S. Diniz B. S. Nicolato R. Kapczinski F. P. Soares J. C. Firmo J. O. (2015). Bipolar disorder prevalence: A systematic review and meta-analysis of the literature. *Rev. Bras. Psiquiatria* 37 155–161. 10.1590/1516-4446-2012-1693 25946396

[B27] Cotovio G. Oliveira-Maia A. J. (2022). Functional neuroanatomy of mania. *Transl. Psychiatry* 12:29. 10.1038/s41398-022-01786-4 35075120 PMC8786958

[B28] Cox M. A. Bassi C. Saunders M. E. Nechanitzky R. Morgado-Palacin I. Zheng C. (2020). Beyond neurotransmission: Acetylcholine in immunity and inflammation. *J. Internal Med.* 287 120–133. 10.1111/joim.13006 31710126

[B29] Cryan J. F. Mombereau C. Vassout A. (2005). The tail suspension test as a model for assessing antidepressant activity: Review of pharmacological and genetic studies in mice. *Neurosci. Biobehav. Rev.* 29, 571–625. 10.1016/j.neubiorev.2005.03.009 15890404

[B30] Dalby-Brown W. Jessen C. Hougaard C. Jensen M. L. Jacobsen T. A. Nielsen K. S. (2013). Characterization of a novel high-potency positive modulator of K(v)7 channels. *Eur. J. Pharmacol.* 709 52–63. 10.1016/j.ejphar.2013.03.039 23562623

[B31] Daniele S. Da Pozzo E. Abelli M. Panighini A. Pini S. Gesi C. (2012). Platelet uptake of GABA and glutamate in patients with bipolar disorder. *Bipolar Disord.* 14 301–308. 10.1111/j.1399-5618.2012.01005.x 22548903

[B32] Daniels S. D. Boison D. (2023). Bipolar mania and epilepsy pathophysiology and treatment may converge in purine metabolism: A new perspective on available evidence. *Neuropharmacology* 241:109756. 10.1016/j.neuropharm.2023.109756 37820933 PMC10841508

[B33] da-Rosa D. D. Valvassori S. S. Steckert A. V. Ornell F. Ferreira C. L. Lopes-Borges J. (2012). Effects of lithium and valproate on oxidative stress and behavioral changes induced by administration of m-AMPH. *Psychiatry Res.* 198 521–526. 10.1016/j.psychres.2012.01.019 22429481

[B34] de Souza Gomes J. A. de Souza G. C. Berk M. Cavalcante L. M. de Sousa F. C. F. Budni J. (2015). Antimanic-like activity of candesartan in mice: Possible involvement of antioxidant, anti-inflammatory and neurotrophic mechanisms. *Eur. Neuropsychopharmacol.* 25 2086–2097. 10.1016/j.euroneuro.2015.08.005 26321203

[B35] Dean J. Keshavan M. (2017). The neurobiology of depression: An integrated view. *Asian J. Psychiatry* 27 101–111. 10.1016/j.ajp.2017.01.025 28558878

[B36] Demontis F. Serra F. Serra G. (2017). Antidepressant-induced dopamine receptor dysregulation: A valid animal model of manic-depressive illness. *Curr. Neuropharmacol.* 15 417–423. 10.2174/1570159X14666160715165648 28503114 PMC5405612

[B37] Dencker D. Dias R. Pedersen M. L. Husum H. (2008). Effect of the new antiepileptic drug retigabine in a rodent model of mania. *Epilepsy Behav.* 12 49–53. 10.1016/j.yebeh.2007.09.023 18086455

[B38] Dubovsky S. L. (2015). Mania. *Continuum* 21 737–755. 10.1212/01.CON.0000466663.28026.6f 26039851

[B39] Egashira N. Goto Y. Iba H. Kawanaka R. Takahashi R. Taniguchi C. (2021). Kamishoyosan potentiates pentobarbital-induced sleep in socially isolated, ovariectomized mice. *J. Ethnopharmacol.* 281:114585. 10.1016/j.jep.2021.114585 34464703

[B40] Ene H. M. Kara N. Z. Barak N. Reshef Ben-Mordechai T. Einat H. (2016). Effects of repeated asenapine in a battery of tests for anxiety-like behaviours in mice. *Acta Neuropsychiatrica* 28 85–91. 10.1017/neu.2015.53 26357996

[B41] Engelhardt K.-A. Schwarting R. K. W. Woehr M. (2018). Mapping trait-like socio-affective phenotypes in rats through 50-kHz ultrasonic vocalizations. *Psychopharmacology* 235 83–98. 10.1007/s00213-017-4746-y 28971233

[B42] Fallah E. Arman S. Najafi M. Shayegh B. (2016). Effect of tamoxifen and lithium on treatment of acute mania symptoms in children and adolescents. *Iranian J. Child Neurol.* 10 16–25. PMC4885151 27247580

[B43] Ferrari A. J. Baxter A. J. Whiteford H. A. (2011). A systematic review of the global distribution and availability of prevalence data for bipolar disorder. *J. Affect. Disord.* 134 1–13. 10.1016/j.jad.2010.11.007 21131055

[B44] Fink K. B. Göthert M. (2007). 5-HT receptor regulation of neurotransmitter release. *Pharmacol. Rev.* 59 360–417. 10.1124/pr.107.07103 18160701

[B45] Flaisher-Grinberg S. Einat H. (2009). A possible utilization of the mice forced swim test for modeling manic-like increase in vigor and goal-directed behavior. *J. Pharmacol. Toxicol. Methods* 59 141–145. 10.1016/j.vascn.2009.03.003 19341808

[B46] Fountoulakis K. N. Tohen M. Zarate C. A. (2022). Lithium treatment of Bipolar disorder in adults: A systematic review of randomized trials and meta-analyses. *Eur. Neuropsychopharmacol. J. Eur. Coll. Neuropsychopharmacol.* 54 100–115. 10.1016/j.euroneuro.2021.10.003 34980362 PMC8808297

[B47] Frey B. N. Andreazza A. C. Cereser K. M. M. Martins M. R. Valvassori S. S. Reus G. Z. (2006a). Effects of mood stabilizers on hippocampus BDNF levels in an animal model of mania. *LIFE Sci.* 79 281–286. 10.1016/j.lfs.2006.01.002 16460767

[B48] Frey B. N. Martins M. R. Petronilho F. C. Dal-Pizzol F. Quevedo J. Kapczinski F. (2006b). Increased oxidative stress after repeated amphetamine exposure: Possible relevance as a model of mania. *Bipolar Disord.* 8 275–280. 10.1111/j.1399-5618.2006.00318.x 16696830

[B49] Giménez-Palomo A. Guitart-Mampel M. Meseguer A. Borràs R. García-García F. J. Tobías E. (2024). Reduced mitochondrial respiratory capacity in patients with acute episodes of bipolar disorder: Could bipolar disorder be a state-dependent mitochondrial disease? *Acta Psychiatrica Scand.* 149 52–64. 10.1111/acps.13635 38030136

[B50] Goloncser F. Baranyi M. Tod P. Maacz F. Sperlagh B. (2024). P2X7 receptor inhibition alleviates mania-like behavior independently of interleukin-1b. *Iscience* 27:109284. 10.1016/j.isci.2024.109284 38444608 PMC10914489

[B51] Gorgulu Y. Uluturk M. K. Palabiyik O. (2021). Comparison of serum BDNF, IL-1β, IL-6, TNF-α, CRP and leucocyte levels in unipolar mania and bipolar disorder. *Acta Neuropsychiatrica* 33 317–322. 10.1017/neu.2021.25 34462030

[B52] Grunnet M. Strøbæk D. Hougaard C. Christophersen P. (2014). Kv7 channels as targets for anti-epileptic and psychiatric drug-development. *Eur. J. Pharmacol.* 726 133–137. 10.1016/j.ejphar.2014.01.017 24457124

[B53] Gubert C. Andrejew R. Leite C. E. Jacintho Moritz C. E. Scholl J. Figueiro F. (2020). P2X7 purinergic receptor is involved in the pathophysiology of mania: A preclinical study. *Mol. Neurobiol.* 57 1347–1360. 10.1007/s12035-019-01817-0 31729632

[B54] Gubert C. Fries G. R. Pfaffenseller B. Ferrari P. Coutinho-Silva R. Morrone F. B. (2016). Role of P2X7 receptor in an animal model of mania induced by D-amphetamine. *Mol. Neurobiol.* 53 611–620. 10.1007/s12035-014-9031-z 25502294

[B55] Guimarães R. P. Resende M. C. S. Tavares M. M. Belardinelli, de Azevedo C. Ruiz M. C. M. (2024). Construct, face, and predictive validity of Parkinson’s disease rodent models. *Int. J. Mol. Sci.* 25:8971. 10.3390/ijms25168971 39201659 PMC11354451

[B56] Hadamitzky M. Markou A. Kuczenski R. (2011). Extended access to methamphetamine self-administration affects sensorimotor gating in rats. *Behav. Brain Res.* 217 386–390. 10.1016/j.bbr.2010.11.009 21070821 PMC3026444

[B57] Han L. Wang L. Tang S. Yuan L. Wu S. Du X. (2018). ITGB4 deficiency in bronchial epithelial cells directs airway inflammation and bipolar disorder-related behavior. *J. Neuroinflammation* 15:246. 10.1186/s12974-018-1283-5 30170608 PMC6117971

[B58] Hao Y. Ge H. Sun M. Gao Y. (2019). Selecting an appropriate animal model of depression. *Int. J. Mol. Sci.* 20:4827. 10.3390/ijms20194827 31569393 PMC6801385

[B59] Hayley A. C. Shiferaw B. Downey L. A. (2021). Amphetamine-induced alteration to gaze parameters: A novel conceptual pathway and implications for naturalistic behavior. *Prog. Neurobiol.* 199:101929. 10.1016/j.pneurobio.2020.101929 33091542

[B60] He S. Wang Q. Chen L. He Y. J. Wang X. Qu S. (2023). miR-100a-5p-enriched exosomes derived from mesenchymal stem cells enhance the anti-oxidant effect in a Parkinson’s disease model via regulation of Nox4/ROS/Nrf2 signaling. *J. Transl. Med.* 21:747. 10.1186/s12967-023-04638-x 37875930 PMC10594913

[B61] Hedya S. A. Avula A. Swoboda H. D. (eds). (2024). “Lithium toxicity,” in *StatPearls* (Florida: StatPearls Publishing). 29763168

[B62] Heinz D. E. Schöttle V. A. Nemcova P. Binder F. P. Ebert T. Domschke K. (2021). Exploratory drive, fear, and anxiety are dissociable and independent components in foraging mice. *Transl. Psychiatry* 11:318. 10.1038/s41398-021-01458-9 34039953 PMC8155035

[B63] Hellvin T. Sundet K. Aminoff S. R. Andreassen O. A. Melle I. (2013). Social functioning in first contact mania: Clinical and neurocognitive correlates. *Compreh. Psychiatry* 54 432–438. 10.1016/j.comppsych.2012.12.016 23351832

[B64] Ho T. C. S. Chan A. H. Y. Ganesan A. (2020). Thirty years of HDAC inhibitors: 2020 insight and hindsight. *J. Med. Chem.* 63 12460–12484. 10.1021/acs.jmedchem.0c00830 32608981

[B65] Hodes A. Lifschytz T. Rosen H. Ben-Ami H. C. Lichtstein D. (2018). Reduction in endogenous cardiac steroids protects the brain from oxidative stress in a mouse model of mania induced by amphetamine. *Brain Res. Bull.* 137 356–362. 10.1016/j.brainresbull.2018.01.016 29374602

[B66] Hsueh Y.-S. Lin C.-Y. Chiu N.-T. Yang Y. K. Chen P. S. Chang H. H. (2021). Changes in striatal dopamine transporters in bipolar disorder and valproate treatment. *Eur. Psychiatry J. Assoc. Eur. Psychiatrists* 64:e9. 10.1192/j.eurpsy.2021.1 33413711 PMC8057387

[B67] Isozaki T. Komenoi S. Lu Q. Usuki T. Tomokata S. Matsutomo D. (2016). Deficiency of diacylglycerol kinase η induces lithium-sensitive mania-like behavior. *J. Neurochem.* 138 448–456. 10.1111/jnc.13661 27167678

[B68] Iwata M. Ota K. T. Li X.-Y. Sakaue F. Li N. Dutheil S. (2016). Psychological stress activates the inflammasome via release of adenosine triphosphate and stimulation of the purinergic Type 2X7 receptor. *Biol. Psychiatry* 80 12–22. 10.1016/j.biopsych.2015.11.026 26831917

[B69] Jiménez-Fernández S. Gurpegui M. Garrote-Rojas D. Gutiérrez-Rojas L. Carretero M. D. Correll C. U. (2021). Oxidative stress parameters and antioxidants in patients with bipolar disorder: Results from a meta-analysis comparing patients, including stratification by polarity and euthymic status, with healthy controls. *Bipolar Disord.* 23 117–129. 10.1111/bdi.12980 32780547

[B70] Johnson D. E. McIntyre R. S. Mansur R. B. Rosenblat J. D. (2023). An update on potential pharmacotherapies for cognitive impairment in bipolar disorder. *Expert Opin. Pharmacother.* 24 641–654. 10.1080/14656566.2023.2194488 36946229

[B71] Kanazawa L. K. S. Radulski D. R. Pereira G. S. Prickaerts J. Schwarting R. K. W. Acco A. (2021). Andrographolide blocks 50-kHz ultrasonic vocalizations, hyperlocomotion and oxidative stress in an animal model of mania. *J. Psychiatric Res.* 139 91–98. 10.1016/j.jpsychires.2021.05.042 34058655

[B72] Kawano T. Inokuchi J. Eto M. Murata M. Kang J.-H. (2021). Activators and Inhibitors of Protein Kinase C (PKC): Their Applications in Clinical Trials. *Pharmaceutics* 13:1748. 10.3390/pharmaceutics13111748 34834162 PMC8621927

[B73] Keramatian K. Pinto J. V. Schaffer A. Sharma V. Beaulieu S. Parikh S. V. (2022). Clinical and demographic factors associated with delayed diagnosis of bipolar disorder: Data from health outcomes and patient evaluations in bipolar disorder (HOPE-BD) study. *J. Affect. Disord.* 296 506–513. 10.1016/j.jad.2021.09.094 34606817

[B74] Komada M. Takao K. Miyakawa T. (2008). Elevated plus maze for mice. *J. Vis. Exp.* 22:1088. 10.3791/1088 19229173 PMC2762911

[B75] Korkmaz Ş. A. Kızgın S. (2023). Neutrophil/high-density lipoprotein cholesterol (HDL), monocyte/HDL and platelet/HDL ratios are increased in acute mania as markers of inflammation, even after controlling for confounding factors. *Curr. Med. Res. Opin.* 39 1383–1390. 10.1080/03007995.2023.2260302 37725087

[B76] Kristensen L. V. Sandager-Nielsen K. Hansen H. H. (2012). K_v_7 (KCNQ) channel openers normalize central 2-deoxyglucose uptake in a mouse model of mania and increase prefrontal cortical and hippocampal serine-9 phosphorylation levels of GSK3β. *J. Neurochem.* 121 373–382. 10.1111/j.1471-4159.2012.07704.x 22356228

[B77] Lee K. M. Coelho M. A. Sern K. R. Class M. A. Bocz M. D. Szumlinski K. K. (2017). Anxiolytic effects of buspirone and MTEP in the Porsolt forced swim test. *Chronic Stress* 1:2470547017712985. 10.1177/2470547017712985 28884167 PMC5584874

[B78] Leem K. H. Kim S. Kim H. W. Park H. J. (2023). Downregulation of microRNA-330-5p induces manic-like behaviors in REM sleep-deprived rats by enhancing tyrosine hydroxylase expression. *CNS Neurosci. Therapeutics* 29 1525–1536. 10.1111/cns.14121 36794530 PMC10173715

[B79] Li X. Chen B. Zhang D. Wang S. Feng Y. Wu X. (2023). A novel murine model of mania. *Mol. Psychiatry* 28 3044–3054. 10.1038/s41380-023-02037-8 36991130 PMC10615760

[B80] Lima D. D. Cyrino L. A. R. Ferreira G. K. Magro D. D. D. Calegari C. R. Cabral H. (2022). Neuroinflammation and neuroprogression produced by oxidative stress in euthymic bipolar patients with different onset disease times. *Sci. Rep.* 12:16742. 10.1038/s41598-022-21170-y 36202963 PMC9537234

[B81] Liu F.-R. Zhou Y. Wang Y. Huang L.-L. Zhang X. Luo H. (2022). Pedigree-based study to identify GOLGB1 as a risk gene for bipolar disorder. *Transl. Psychiatry* 12:390. 10.1038/s41398-022-02163-x 36115840 PMC9482626

[B82] Lopachev A. Volnova A. Evdokimenko A. Abaimov D. Timoshina Y. Kazanskaya R. (2019). Intracerebroventricular injection of ouabain causes mania-like behavior in mice through D2 receptor activation. *Sci. Rep.* 9:15627. 10.1038/s41598-019-52058-z 31666560 PMC6821712

[B83] Lutfy R. H. Essawy A. E. Mohammed H. S. Shakweer M. M. Salam S. A. (2024). Transcranial irradiation mitigates paradoxical sleep deprivation effect in an age-dependent manner: Role of BDNF and GLP-1. *Neurochem. Res.* 49 919–934. 10.1007/s11064-023-04071-y 38114728 PMC10902205

[B84] Malhi G. S. Tanious M. Das P. Coulston C. M. Berk M. (2013). Potential mechanisms of action of lithium in bipolar disorder. Current understanding. *CNS Drugs* 27 135–153. 10.1007/s40263-013-0039-0 23371914

[B85] Maniglio R. (2013). Prevalence of child sexual abuse among adults and youths with bipolar disorder: A systematic review. *Clin. Psychol. Rev.* 33 561–573. 10.1016/j.cpr.2013.03.002 23563080

[B86] Mannangatti P. Ramamoorthy S. Jayanthi L. D. (2018). Interference of norepinephrine transporter trafficking motif attenuates amphetamine-induced locomotor hyperactivity and conditioned place preference. *Neuropharmacology* 128 132–141. 10.1016/j.neuropharm.2017.10.005 28986281 PMC5714664

[B87] Martino M. Magioncalda P. Huang Z. Conio B. Piaggio N. Duncan N. W. (2016). Contrasting variability patterns in the default mode and sensorimotor networks balance in bipolar depression and mania. *Proc. Natl. Acad. Sci. U S A.* 113 4824–4829. 10.1073/pnas.1517558113 27071087 PMC4855585

[B88] Menegas S. Dal-Pont G. C. Cararo J. H. Varela R. B. Aguiar-Geraldo J. M. Possamai-Della T. (2020). Efficacy of folic acid as an adjunct to lithium therapy on manic-like behaviors, oxidative stress and inflammatory parameters in an animal model of mania. *Metabolic Brain Dis.* 35 413–425. 10.1007/s11011-019-00503-3 31840201

[B89] Menegas S. Ferreira C. L. Cararo J. H. Gava F. F. Dal-Pont G. C. Gomes M. L. (2019). Resveratrol protects the brain against oxidative damage in a dopaminergic animal model of mania. *Metabolic Brain Dis.* 34 941–950. 10.1007/s11011-019-00408-1 30919245

[B90] Miola A. Dal Porto V. Meda N. Perini G. Solmi M. Sambataro F. (2022). Secondary Mania induced by TNF-α inhibitors: A systematic review. *Psychiatry Clin. Neurosci.* 76 15–21. 10.1111/pcn.13302 34590391 PMC9298409

[B91] Mitchell A. Heales L. Treleaven J. Too B. Tyrrell R. Dinsdale A. (2024). Pain-free bite force in a healthy population: Within-session test-retest reliability in different sitting positions. *J. Oral Rehabil.* 51 1440–1449. 10.1111/joor.13720 38685714

[B92] Miyake N. Thompson J. Skinbjerg M. Abi-Dargham A. (2011). Presynaptic dopamine in schizophrenia. *CNS Neurosci. Therapeutics* 17 104–109. 10.1111/j.1755-5949.2010.00230.x 21199451 PMC6493810

[B93] Mondejar-Parreño G. Perez-Vizcaino F. Cogolludo A. (2020). Kv7 channels in lung diseases. *Front. Physiol.* 11:634. 10.3389/fphys.2020.00634 32676036 PMC7333540

[B94] Moretti M. Valvassori S. S. Varela R. B. Ferreira C. L. Rochi N. Benedet J. (2011). Behavioral and neurochemical effects of sodium butyrate in an animal model of mania. *Behav. Pharmacol.* 22 766–772. 10.1097/FBP.0b013e32834d0f1b 21989497

[B95] Morton E. Murray G. Yatham L. N. Lam R. W. Michalak E. E. (2021). The Quality of Life in Bipolar Disorder (QoL.BD) questionnaire a decade on—A systematic review of the measurement of condition-specific aspects of quality of life in bipolar-disorder. *J. Affect. Disord.* 278 33–45. 10.1016/j.jad.2020.09.017 32949871

[B96] Mullins N. Forstner A. J. O’Connell K. S. Coombes B. Coleman J. R. I. Qiao Z. (2021). Genome-wide association study of more than 40,000 bipolar disorder cases provides new insights into the underlying biology. *Nat. Genet.* 53, 817–829. 10.1038/s41588-021-00857-4IF:31.7 34002096 PMC8192451

[B97] Noda M. (2016). Dysfunction of glutamate receptors in microglia may cause neurodegeneration. *Curr. Alzheimer Res.* 13 381–386. 10.2174/1567205013666151116125810 26567741

[B98] Owens W. A. Williams J. M. Saunders C. Avison M. J. Galli A. Daws L. C. (2012). Rescue of dopamine transporter function in hypoinsulinemic rats by a D2 receptor-ERK-dependent mechanism. *J. Neurosci.* 32 2637–2647. 10.1523/JNEUROSCI.3759-11.2012 22357848 PMC3310897

[B99] Park J.-I. (2023). MAPK-ERK pathway. *Int. J. Mol. Sci.* 24:9666. 10.3390/ijms24119666 37298618 PMC10253477

[B100] Pentkowski N. S. Rogge-Obando K. K. Donaldson T. N. Bouquin S. J. Clark B. J. (2021). Anxiety and Alzheimer’s disease: Behavioral analysis and neural basis in rodent models of Alzheimer’s-related neuropathology. *Neurosci. Biobehav. Rev.* 127, 647–658. 10.1016/j.neubiorev.2021.05.005 33979573 PMC8292229

[B101] Pereira A. C. Oliveira J. Silva S. Madeira N. Pereira C. M. F. Cruz M. T. (2021). Inflammation in bipolar disorder (BD): Identification of new therapeutic targets. *Pharmacol. Res.* 163:105325. 10.1016/j.phrs.2020.105325 33278569

[B102] Petroff O. A. C. (2002). GABA and glutamate in the human brain. *Neurosci. Rev. J. Bringing Neurobiol. Neurol. Psychiatry* 8 562–573. 10.1177/1073858402238515 12467378

[B103] Phan D. Shin E. Jeong J. H. Tran H. Sharma N. Nguyen B. T. (2020). Lithium attenuates d-amphetamine-induced hyperlocomotor activity in mice via inhibition of interaction between cyclooxygenase-2 and indoleamine-2,3-dioxygenase. *Clin. Exp. Pharmacol. Physiol.* 47 790–797. 10.1111/1440-1681.13243 31883280

[B104] Primo M. J. Fonseca-Rodrigues D. Almeida A. Teixeira P. M. Pinto-Ribeiro F. (2023). Sucrose preference test: A systematic review of protocols for the assessment of anhedonia in rodents. *Eur. Neuropsychopharmacol. J. Eur. Coll. Neuropsychopharmacol.* 77 80–92. 10.1016/j.euroneuro.2023.08.496 37741164

[B105] Qiu X.-M. Sun Y.-Y. Ye X.-Y. Li Z.-G. (2019). Signaling role of glutamate in plants. *Front. Plant Sci.* 10:1743. 10.3389/fpls.2019.01743 32063909 PMC6999156

[B106] Rihmer Z. Gonda X. Döme P. (2017). Is mania the hypertension of the mood? *Discussion A Hypothesis. Curr. Neuropharmacol.* 15 424–433. 10.2174/1570159X14666160902145635 28503115 PMC5405605

[B107] Rippberger H. van Gaalen M. M. Schwarting R. K. W. Wohr M. (2015). Environmental and pharmacological modulation of amphetamine- induced 50-kHz ultrasonic vocalizations in rats. *Curr. Neuropharmacol.* 13 220–232. 10.2174/1570159x1302150525124408 26411764 PMC4598433

[B108] Sakrajda K. Szczepankiewicz A. (2021). Inflammation-Related Changes in Mood Disorders and the Immunomodulatory Role of Lithium. *Int. J. Mol. Sci.* 22:1532. 10.3390/ijms22041532 33546417 PMC7913492

[B109] Sandoval-Sánchez A. R. Cedillo Zavaleta L. N. Jiménez J. C. Ruíz-García I. Miranda F. (2020). Administration of low doses of the 5-HT1A receptor agonist 8-OH-DPAT attenuates the discriminative signal of amphetamine in the conditioned taste aversion procedure. *Pharmacol. Biochem. Behav.* 193:172932. 10.1016/j.pbb.2020.172932 32315693

[B110] Scotti-Muzzi E. Chile T. Moreno R. Pastorello B. F. da Costa Leite C. (2021). ACC Glu/GABA ratio is decreased in euthymic bipolar disorder I patients: Possible in vivo neurometabolite explanation for mood stabilization. *Eur. Arch. Psychiatry Clin. Neurosci.* 271 537–547. 10.1007/s00406-020-01096-0 31993746

[B111] Shiah I. S. Yatham L. N. (2000). Serotonin in mania and in the mechanism of action of mood stabilizers: A review of clinical studies. *Bipolar Disord.* 2 77–92. 10.1034/j.1399-5618.2000.020201.x 11252655

[B112] Shin E.-J. Duy-Khanh D. Hwang Y. G. Hai-Quyen T. Sharma N. Jeong J. H. (2019). Significance of protein kinase C in the neuropsychotoxicity induced by methamphetamine-like psychostimulants. *Neurochem. Int.* 124 162–170. 10.1016/j.neuint.2019.01.014 30654115

[B113] Shoblock J. R. Sullivan E. B. Maisonneuve I. M. Glick S. D. (2003). Neurochemical and behavioral differences between d-methamphetamine and d-amphetamine in rats. *Psychopharmacology* 165 359–369. 10.1007/s00213-002-1288-7 12491026

[B114] Siefried K. J. Acheson L. S. Lintzeris N. Ezard N. (2020). Pharmacological treatment of methamphetamine/amphetamine dependence: A systematic review. *CNS Drugs* 34, 337–365. 10.1007/s40263-020-00711-x 32185696 PMC7125061

[B115] Sies H. (2015). Oxidative stress: A concept in redox biology and medicine. *Redox Biol.* 4 180–183. 10.1016/j.redox.2015.01.002 25588755 PMC4309861

[B116] Snyder S. H. Innis R. B. (1979). Peptide neurotransmitters. *Annu. Rev. Biochem.* 48, 755–782. 10.1146/annurev.bi.48.070179.003543 38738

[B117] Souza L. S. Silva E. F. Santos W. B. Asth L. Lobão-Soares B. Soares-Rachetti V. P. (2016). Lithium and valproate prevent methylphenidate-induced mania-like behaviors in the hole board test. *Neurosci. Lett.* 629 143–148. 10.1016/j.neulet.2016.06.044 27353513

[B118] Stautland A. Jakobsen P. Fasmer O. B. Osnes B. Torresen J. Nordgreen T. (2023). Reduced heart rate variability during mania in a repeated naturalistic observational study. *Front. Psychiatry* 14:1250925. 10.3389/fpsyt.2023.1250925 37743991 PMC10513449

[B119] Steckert A. V. Valvassori S. S. Varela R. B. Mina F. Resende W. R. Bavaresco D. V. (2013). Effects of sodium butyrate on oxidative stress and behavioral changes induced by administration of D-AMPH. *Neurochem. Int.* 62 425–432. 10.1016/j.neuint.2013.02.001 23411414

[B120] Stertz L. Fries G. R. de Aguiar B. W. Pfaffenseller B. Valvassori S. S. Gubert C. (2014). Histone deacetylase activity and brain-derived neurotrophic factor (BDNF) levels in a pharmacological model of mania. *Rev. Brasileira Psiquiatria* 36 39–46. 10.1590/1516-4446-2013-1094 24346357

[B121] Støier J. F. Konomi-Pilkati A. Apuschkin M. Herborg F. Gether U. (2023). Amphetamine-induced reverse transport of dopamine does not require cytosolic Ca2. *J. Biol. Chem.* 299:105063. 10.1016/j.jbc.2023.105063 37468107 PMC10448275

[B122] Sun X.-Q. Peters E. L. Schalij I. Axelsen J. B. Andersen S. Kurakula K. (2021). Increased MAO-A activity promotes progression of pulmonary arterial hypertension. *Am. J. Respiratory Cell Mol. Biol.* 64 331–343. 10.1165/rcmb.2020-0105OC 33264068

[B123] Tan X. Qi C. Zhao X. Sun L. Wu M. Sun W. (2023). ERK inhibition promotes engraftment of allografts by reprogramming T-cell metabolism. *Adv. Sci.* 10:e2206768. 10.1002/advs.202206768 37013935 PMC10238213

[B124] Tondo L. Vázquez G. H. Baldessarini R. J. (2017). Depression and mania in bipolar disorder. *Curr. Neuropharmacol.* 15 353–358. 10.2174/1570159X14666160606210811 28503106 PMC5405618

[B125] Tran H.-Q. Shin E.-J. Saito K. Tran T.-V. Phan D.-H. Sharma N. (2020). Indoleamine-2,3-dioxygenase-1 is a molecular target for the protective activity of mood stabilizers against mania-like behavior induced by d-amphetamine. *Food Chem. Toxicol.* 136:110986. 10.1016/j.fct.2019.110986 31760073

[B126] Trevizol F. Benvegnu D. M. Barcelos R. C. S. Boufleur N. Dolci G. S. Mueller L. G. (2011). Comparative study between n-6, *trans* and n-3 fatty acids on repeated amphetamine exposure: A possible factor for the development of mania. *Pharmacol. Biochem. Behav.* 97 560–565. 10.1016/j.pbb.2010.11.004 21078338

[B127] Trunnell E. R. Carvalho C. (2021). The forced swim test has poor accuracy for identifying novel antidepressants. *Drug Discov. Today* 26 2898–2904. 10.1016/j.drudis.2021.08.003 34390862

[B128] Underhill S. M. Colt M. S. Amara S. G. (2020). Amphetamine stimulates endocytosis of the norepinephrine and neuronal glutamate transporters in cultured locus coeruleus neurons. *Neurochem. Res.* 45 1410–1419. 10.1007/s11064-019-02939-6 31912366 PMC7260265

[B129] Valvassori S. S. Aguiar-Geraldo J. M. Possamai-Della T. da-Rosa D. D. Peper-Nascimento J. Cararo J. H. (2022). Depressive-like behavior accompanies neuroinflammation in an animal model of bipolar disorder symptoms induced by ouabain. *Pharmacol. Biochem. Behav.* 219:173434. 10.1016/j.pbb.2022.173434 35901967

[B130] Valvassori S. S. Calixto K. V. Budni J. Resende W. R. Varela R. B. de Freitas K. V. (2013). Sodium butyrate reverses the inhibition of Krebs cycle enzymes induced by amphetamine in the rat brain. *J. Neural Transmission* 120 1737–1742. 10.1007/s00702-013-1056-3 23851624

[B131] Valvassori S. S. Elias G. de Souza B. Petronilho F. Dal-Pizzol F. Kapczinski F. (2011). Effects of cannabidiol on amphetamine-induced oxidative stress generation in an animal model of mania. *J. Psychopharmacol.* 25 274–279. 10.1177/0269881109106925 19939866

[B132] Valvassori S. S. Gava F. F. Dal-Pont G. C. Simoes H. L. Damiani-Neves M. Andersen M. L. (2019a). Effects of lithium and valproate on ERK/JNK signaling pathway in an animal model of mania induced by amphetamine. *Heliyon* 5:e01541. 10.1016/j.heliyon.2019.e01541 31193305 PMC6525279

[B133] Valvassori S. S. Mariot E. Varela R. B. Bavaresco D. V. Dal-Pont G. C. Ferreira C. L. (2019b). The role of neurotrophic factors in manic-, anxious- and depressive-like behaviors induced by amphetamine sensitization: Implications to the animal model of bipolar disorder. *J. Affect. Disord.* 245 1106–1113. 10.1016/j.jad.2018.10.370 30699853

[B134] Valvassori S. S. Petronilho F. C. Réus G. Z. Steckert A. V. Oliveira V. B. Boeck C. R. (2008). Effect of N-acetylcysteine and/or deferoxamine on oxidative stress and hyperactivity in an animal model of mania. *Prog. Neuro-psychopharmacol. Biol. Psychiatry* 32, 1064–1068. 10.1016/j.pnpbp.2008.02.012IF:5.3 18403082

[B135] Valvassori S. S. Tonin P. T. Dal-Pont G. C. Varela R. B. Cararo J. H. Garcia A. F. (2019c). Coadministration of lithium and celecoxib reverses manic-like behavior and decreases oxidative stress in a dopaminergic model of mania induced in rats. *Transl. Psychiatry* 9:297. 10.1038/s41398-019-0637-9 31723123 PMC6853972

[B136] Valvassori S. S. Tonin P. T. Varela R. B. Carvalho A. F. Mariot E. Amboni R. T. (2015). Lithium modulates the production of peripheral and cerebral cytokines in an animal model of mania induced by dextroamphetamine. *Bipolar Disord.* 17 507–517. 10.1111/bdi.12299 25929806

[B137] van Enkhuizen J. Janowsky D. S. Olivier B. Minassian A. Perry W. Young J. W. (2015a). The catecholaminergic-cholinergic balance hypothesis of bipolar disorder revisited. *Eur. J. Pharmacol.* 753 114–126. 10.1016/j.ejphar.2014.05.063 25107282 PMC4318788

[B138] van Enkhuizen J. Milienne-Petiot M. Geyer M. A. Young J. W. (2015b). Modeling bipolar disorder in mice by increasing acetylcholine or dopamine: Chronic lithium treats most, but not all features. *Psychopharmacology* 232 3455–3467. 10.1007/s00213-015-4000-4 26141192 PMC4537820

[B139] Varela R. B. Boschen S. L. Yates N. Houghton T. Blaha C. D. Lee K. H. (2024). Anti-manic effect of deep brain stimulation of the ventral tegmental area in an animal model of mania induced by methamphetamine. *Bipolar Disord.* 26 376–387. 10.1111/bdi.13423 38558302

[B140] Varela R. B. Resende W. R. Dal-Pont G. C. Gava F. F. Tye S. J. Quevedo J. (2020). HDAC inhibitors reverse mania-like behavior and modulate epigenetic regulatory enzymes in an animal model of mania induced by Ouabain. *Pharmacol. Biochem. Behav.* 193:172917. 10.1016/j.pbb.2020.172917 32222371

[B141] Varela R. B. Valvassori S. S. Lopes-Borges J. Fraga D. B. Resende W. R. Arent C. O. (2013). Evaluation of acetylcholinesterase in an animal model of mania induced by D-amphetamine. *Psychiatry Res.* 209 229–234. 10.1016/j.psychres.2012.11.021 23245536

[B142] Vargas K. M. Da Cunha C. Andreatini R. (2006). Amphetamine and pentylenetetrazole given post-trial 1 enhance one-trial tolerance to the anxiolytic effect of diazepam in the elevated plus-maze in mice. *Prog. Neuro Psychopharmacol. Biol. Psychiatry* 30 1394–1402. 10.1016/j.pnpbp.2006.05.004 16828217

[B143] Verharen J. P. H. de Jong J. W. Zhu Y. Lammel S. (2023). A computational analysis of mouse behavior in the sucrose preference test. *Nat. Commun.* 14:2419. 10.1038/s41467-023-38028-0 37105954 PMC10140068

[B144] Volkow N. D. Michaelides M. Baler R. (2019). The neuroscience of drug reward and addiction. *Physiol. Rev.* 99, 2115–2140. 10.1152/physrev.00014.2018 31507244 PMC6890985

[B145] Walz J. C. Frey B. N. Andreazza A. C. Cereser K. M. Cacilhas A. A. Valvassori S. S. (2008). Effects of lithium and valproate on serum and hippocampal neurotrophin-3 levels in an animal model of mania. *J. Psychiatric Res.* 42 416–421. 10.1016/j.jpsychires.2007.03.005 17512948

[B146] Waples R. S. (2022). What is ne. Anyway? *J. Heredity* 113 371–379. 10.1093/jhered/esac023 35532202

[B147] Yan C. Yang Z. Chen P. Yeh Y. Sun C. Xie T. (2024). GPR65 sensing tumor-derived lactate induces HMGB1 release from TAM via the cAMP/PKA/CREB pathway to promote glioma progression. *J. Exp. Clin. Cancer Res.* 43:105. 10.1186/s13046-024-03025-8 38576043 PMC10993467

[B148] Yan H. C. Cao X. Das M. Zhu X. H. Gao T. M. (2010). Behavioral animal models of depression. *Neurosci. Bull.* 26, 327–337. 10.1007/s12264-010-0323-7 20651815 PMC5552573

[B149] Yang L. Ma X. Guo Y. He Y. Yang Y. Wang W. (2023). Acetylcholine (ACh) enhances Cd tolerance through transporting ACh in vesicles and modifying Cd absorption in duckweed (Lemna turionifera 5511). *Environ. Pollut.* 335:122305. 10.1016/j.envpol.2023.122305 37580008

[B150] Yankelevitch-Yahav R. Franko M. Huly A. Doron R. (2015). The forced swim test as a model of depressive-like behavior. *J. Vis. Exp.* 97:52587. 10.3791/52587 25867960 PMC4401172

[B151] Yap L.-P. Sancheti H. Ybanez M. D. Garcia J. Cadenas E. Han D. (2010). Determination of GSH, GSSG, and GSNO using HPLC with electrochemical detection. *Methods Enzymol.* 473 137–147. 10.1016/S0076-6879(10)73006-8 20513475 PMC3040068

[B152] Ye D. Xu H. Tang Q. Xia H. Zhang C. Bi F. (2021). The role of 5-HT metabolism in cancer. *Biochim. Biophys. Acta Rev. Cancer* 1876:188618. 10.1016/j.bbcan.2021.188618 34428515

[B153] Yin H.-S. Lai C.-C. Tien T.-W. Han S.-K. Pu X.-L. (2010). Differential changes in cerebellar transmitter content and expression of calcium binding proteins and transcription factors in mouse administered with amphetamine. *Neurochem. Int.* 57 288–296. 10.1016/j.neuint.2010.06.007 20600441

[B154] Yoo J. H. Ha T.-W. Hong J. T. Oh K.-W. (2017). Sinomenine, an alkaloid derived from *Sinomenium acutum* potentiates pentobarbital-induced sleep behaviors and non-rapid eye movement (NREM) sleep in rodents. *Biomol. Therapeutics* 25 586–592. 10.4062/biomolther.2017.157 29081090 PMC5685427

[B155] Young J. W. Henry B. L. Geyer M. A. (2011). Predictive animal models of mania: Hits, misses and future directions. *Br. J. Pharmacol.* 164 1263–1284. 10.1111/j.1476-5381.2011.01318.x 21410454 PMC3229761

[B156] Yu Q. Wang X. Li X. Bai X. Zhao R. Peng X. (2023). Purinergic P2X7R as a potential target for pancreatic cancer. *Clin. Transl. Oncol.* 25 2297–2305. 10.1007/s12094-023-03123-7 36856920

[B157] Zald D. H. (2023). The influence of dopamine autoreceptors on temperament and addiction risk. *Neurosci. Biobehav. Rev.* 155:105456. 10.1016/j.neubiorev.2023.105456 37926241 PMC11330662

[B158] Zarate C. A. Singh J. B. Carlson P. J. Quiroz J. Jolkovsky L. Luckenbaugh D. A. (2007). Efficacy of a protein kinase C inhibitor (tamoxifen) in the treatment of acute mania: A pilot study. *Bipolar Disord.* 9 561–570. 10.1111/j.1399-5618.2007.00530.x 17845270

[B159] Zeng W. Song Y. Wang R. He R. Wang T. (2023). Neutrophil elastase: From mechanisms to therapeutic potential. *J. Pharm. Anal.* 13 355–366. 10.1016/j.jpha.2022.12.003 37181292 PMC10173178

[B160] Zestos A. G. Carpenter C. Kim Y. Low M. J. Kennedy R. T. Gnegy M. E. (2019). Ruboxistaurin reduces cocaine-stimulated increases in extracellular dopamine by modifying dopamine-autoreceptor activity. *ACS Chem. Neurosci.* 10 1960–1969. 10.1021/acschemneuro.8b00259 30384585 PMC6470047

[B161] Zhang L. Cheng D. Zhang J. Tang H. Li F. Peng Y. (2023). Role of macrophage AHR/TLR4/STAT3 signaling axis in the colitis induced by non-canonical AHR ligand aflatoxin B1. *J. Hazardous Mater.* 452:131262. 10.1016/j.jhazmat.2023.131262 36989784

[B162] Zhang Y. Zhang Z. Zhang Y. Wu L. Gao L. Yao R. (2022). Baicalin promotes the activation of brown and white adipose tissue through AMPK/PGC1? pathway. *Eur. J. Pharmacol.* 922:174913. 10.1016/j.ejphar.2022.174913 35337814

